# Dealing With Missing, Imbalanced, and Sparse Features During the Development of a Prediction Model for Sudden Death Using Emergency Medicine Data: Machine Learning Approach

**DOI:** 10.2196/38590

**Published:** 2023-01-20

**Authors:** Xiaojie Chen, Han Chen, Shan Nan, Xiangtian Kong, Huilong Duan, Haiyan Zhu

**Affiliations:** 1 Key Laboratory of Biomedical Engineering of Hainan Province School of Biomedical Engineering Hainan University Haikou China; 2 Hainan Hospital of Chinese People's Liberation Army General Hospital Sanya China; 3 IMWare Wuhan China; 4 College of Biomedical Engineering and Instrumental Science Zhejiang University Hangzhou China; 5 First Medical Center of Chinese People's Liberation Army General Hospital Beijing China

**Keywords:** emergency medicine, prediction model, data preprocessing, imbalanced data, missing value interpolation, sparse features, clinical informatics, machine learning, medical informatics

## Abstract

**Background:**

In emergency departments (EDs), early diagnosis and timely rescue, which are supported by prediction modes using ED data, can increase patients’ chances of survival. Unfortunately, ED data usually contain missing, imbalanced, and sparse features, which makes it challenging to build early identification models for diseases.

**Objective:**

This study aims to propose a systematic approach to deal with the problems of missing, imbalanced, and sparse features for developing sudden-death prediction models using emergency medicine (or ED) data.

**Methods:**

We proposed a 3-step approach to deal with data quality issues: a random forest (RF) for missing values, k-means for imbalanced data, and principal component analysis (PCA) for sparse features. For continuous and discrete variables, the decision coefficient R^2^ and the κ coefficient were used to evaluate performance, respectively. The area under the receiver operating characteristic curve (AUROC) and the area under the precision-recall curve (AUPRC) were used to estimate the model’s performance. To further evaluate the proposed approach, we carried out a case study using an ED data set obtained from the Hainan Hospital of Chinese PLA General Hospital. A logistic regression (LR) prediction model for patient condition worsening was built.

**Results:**

A total of 1085 patients with rescue records and 17,959 patients without rescue records were selected and significantly imbalanced. We extracted 275, 402, and 891 variables from laboratory tests, medications, and diagnosis, respectively. After data preprocessing, the median R^2^ of the RF continuous variable interpolation was 0.623 (IQR 0.647), and the median of the κ coefficient for discrete variable interpolation was 0.444 (IQR 0.285). The LR model constructed using the initial diagnostic data showed poor performance and variable separation, which was reflected in the abnormally high odds ratio (OR) values of the 2 variables of cardiac arrest and respiratory arrest (201568034532 and 1211118945, respectively) and an abnormal 95% CI. Using processed data, the recall of the model reached 0.746, the *F*_1_-score was 0.73, and the AUROC was 0.708.

**Conclusions:**

The proposed systematic approach is valid for building a prediction model for emergency patients.

## Introduction

In the emergency department (ED), early identification of high-risk patients can improve clinical decisions, avoid waste of resources, and lead to better patient prognosis [1,2]. A prospective study showed that the incidence of adverse events due to improper emergency care is about 5%-10%, of which half can be prevented through early detection [3]. However, early identification is difficult as these patients often show little obvious signs before rapid deterioration [4].

Prediction models for high-risk patients in EDs can greatly support caregivers [5]. Electronic medical record (EMR) data, which fully capture patients’ status, are an important source for developing disease risk prediction models [6]. As a typical high-risk disease in EDs, sudden death is a major public health problem worldwide, accounting for 15%-20% of all deaths [7,8]. A previous study showed that cardiogenic diseases, potassium, mean platelet volume, creatinine, chloride, and sodium are important variables to predict the risk of death in patients [5]. A survey showed that age, male, hypertension, diabetes, hypercholesterolemia, and a family history of coronary heart disease are all associated with increased risk of sudden death [9]. A study evaluating the relationship between the variables of laboratory tests and the occurrence of acute death in patients found that serum sodium, glucose, and the leukocyte count show a U-shaped relationship with mortality [10]. In addition, total bilirubin, creatine kinase, the international normalized ratio, aspartate aminotransferase, and lactate dehydrogenase are all risk factors associated with acute death in patients [11-13]. However, the data quality of EMRs limits their effective use for developing prediction models [6,14]. Prediction of sudden death needs a variety of clinical data, which are frequently missing, imbalanced, and having sparse features.

Missing values, imbalanced data, and sparse features are 3 common problems of EMR data. Missing values indicate not enough data collected due to improper use of the hospital information system or other reasons [14]. Imbalanced data refer to the imbalanced distribution of negative and positive samples. This leads to more features of negative samples in the learning model, which is not suitable for the prediction of arbitrary patients [15,16]. Sparse features are zero features that are much larger than nonzero features and increase computing memory and reduce generalization ability [17,18]. Especially in small samples, a large amount of noise in sparse features makes model training impossible to converge. Therefore, tackling these quality issues of EMR data is an essential step to improve the predictive performance of machine learning (ML) models.

To solve the aforementioned 3 problems, we propose a series of ML approaches to increase fitting ability and generalization ability. Using the approach, we developed a sudden-death predication model. The risk factors related to sudden death obtained through logistic regression (LR) model were consistent with the results reported in the earlier literature on the analysis of risk factors of in-hospital death. These results show that our data-preprocessing approach can effectively maintain the rich information contained in emergency data and provide a reliable data source for the development of a sudden-death prediction model.

## Methods

### Study Design

**Figure 1:**
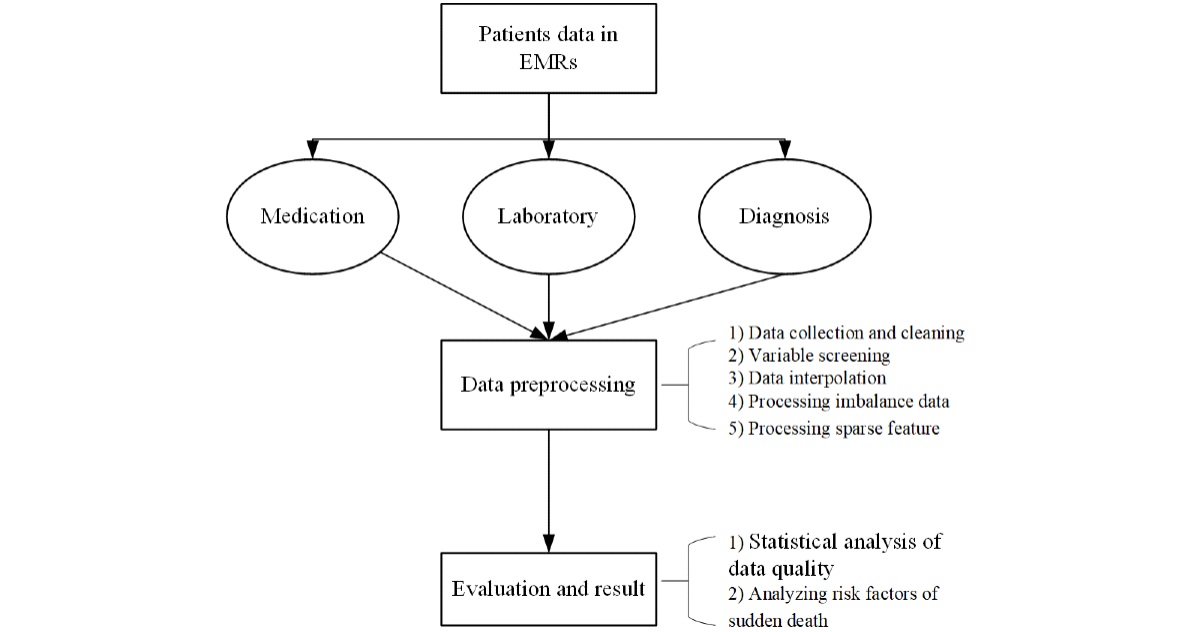
Workflow of ED data preprocessing and evaluation. ED: emergency department; EMR: electronic medical record.

Our methods of data preprocessing consisted of 5 steps, as shown in Figure 1. The last 3 steps tackle 3 low-quality issues: missing values, imbalanced data, and sparse features. Finally, postprocessing data quality is evaluated by a sudden-death prediction model case study.

### Data Collection and Cleaning

Data for ED patient prediction model development are summarized in Table 1.

Close investigation of each data table is required so as to know the location of our content of interest. For instance, data regarding a patient’s basic information are stored in the emg_visit table. Lab test items and results are stored in the lab_result and lab_master tables. The clinical record field in the emg_order table can be used to determine whether a sudden-death event occurred. One lab test (eg, blood test) can be performed multiple times to observe the patient status closely. Based on clinical experts’ opinions, only the last one is meaningful.

**Table 1:** Description of the data table involved in the query process.

Table name	Data description
emg_drug detail	The patient's medication record, including the prescription number, drug name, dosage, drug specification, administration time, and administration route during the treatment period
emg_drug_master	Master record form of patient medication recording patient ID and prescription number
emg_order	Doctor’s order record form used to record the medication, inspection, diagnosis, treatment, and other doctor’s orders of the patient during treatment
emg_visit	Patient visit information table, including the patient's basic personal information, diagnosis of the current visit, triage, and other information
lab_test_master	Patient’s laboratory test master record form recording the patient’s age and gender information, laboratory test items made during the visit, and the corresponding doctor’s order ID
lab_result	Laboratory test results of patients, including test results of patients

### Variable Screening

The number of variables obtained from the data collection was large, so screening of important variables facilitated final analysis. Two approaches can be adopted. One is based on statistical significance. The other is based on the specific research objective, opinions of medical experts, or authoritative literature [5,12,13]. In our study, the first approach was taken. Variables with many missing values were filtered out using the threshold. For example, Alvarez et al [19] set the threshold to 2%, while Seki et al [20] set it to 25%. In this study, we set the threshold to 80%. This means that when 80% of the values of 1 variable are missing, that variable should be filtered out.

### Data Interpolation for Missing Values

Missing values affect the effectiveness of ML models. Data missing show 3 different patterns: missing completely at random (MCAR), missing at random deletion (MAR), and not missing at random (MNAR). MCAR means that the missing of data is completely random and does not depend on observed or unobserved values [21]. In this case, any interpolation method will not cause deviation. However, the assumption of MCAR in actual data is difficult to satisfy [22,23]. MNAR and MAR mean that the missing of data depends on the unobserved value and does not depend on the unobserved value, respectively [24]. However, it is impossible to infer whether the missing pattern belongs to MNAR or MAR through the existing data containing the missing pattern, and the assumption based on MAR is more consistent with the actual data situation [22,25]. MAR allows us to estimate missing values using existing observation data in the data set [24].

The goal of all kinds of interpolation methods is to reasonably estimate missing values and improve the quality of data. Interpolation methods are mainly divided into single interpolation and multiple interpolation. Multiple interpolation is a commonly used and better performance interpolation method. It generates multiple possible estimates for missing data and uses statistical inference to interpolate the final value. This method can reflect the randomness of missing data, and the interpolation error is smaller [21]. In a single interpolation, interpolation methods, such as constants (ie, specific identifications), mean, median, and data distribution, can be used. However, such methods usually cause greater deviation [26,27]. The single interpolation method based on ML has attracted increasingly more attention [23], such as interpolation based on a clustering algorithm [28], an ensemble model [29], and Bayesian theory [30]. Although multiple imputation can bring smaller deviation, when the frequency of missing data is high and the sample size is small, multiple imputation should be considered [31]. However, its implementation is relatively complex, and it needs to involve the selection of an interpolation model and the number of interpolation data created [32]. When the data are sufficient and the variability of the estimated value does not need to be considered, it is feasible to choose multiple imputation or single imputation [31]. Considering that our sample size was relatively sufficient, to build a simpler interpolation method, we used a random forest (RF) [33,34] as the interpolation algorithm to realize the interpolation of missing data in the form of a single interpolation.

Altogether, the followed steps are proposed.

- For variable “i,” 1 set of patient samples without missing values work as training samples and the other set of patient samples with missing values work as test samples.
- If other variables in the 2 samples are missing, the mean (continuous variable) or mode (discrete variable) is temporarily interpolated to form a complete sample.
- Use training samples to train RF models, the model is applied to test samples to predict missing values.
- For the next variable, steps 1, 2, and 3 are repeated until all variables of the whole sample are interpolated.

### Processing Imbalanced Data

Imbalanced data refer to the imbalanced distribution of negative and positive samples. For example, in the classification of rare diseases and credit predictions, there could be more negative samples than positive ones. Because most ML algorithms assume that categories (eg, positive or negative) of samples are evenly distributed, classifying models trained with imbalanced data are more likely to classify a new sample into the majority category [15].

Basic solutions for imbalanced data are to use under- or oversampling to make the data balanced, such as random oversampling [35], random undersampling, the synthetic minority oversampling technique (SMOTE) [36], and the adaptive synthetic sampling method (ADASYN) [15]. Although both undersampling and oversampling approaches can achieve data balance, the oversampling approach adds many sample copies to overfit the model. Wang and Japkowicz [16] and Chawla et al [36] also argued that undersampling is more favorable than oversampling in extreme imbalance situations. However, randomly discarding undersampling may also lose some representative samples. Segura-Bedmar et al [37] and Lin et al [38] proposed a clustering method to tackle this problem. The k-means considers the similarity between samples and uses the sample closest to the centroid of the cluster to approximate all the sample characteristics within the cluster, and the obtained samples are representative. The advantage of the clustering method over random undersampling is that all samples are used in the clustering process. This ensures that the information about all samples can be used to determine the sampling results and some important samples are not randomly discarded. In addition, we can adjust the number of clusters in k-means according to the actual data imbalance so as to achieve different undersampling ratios without other complex adjustments.

To avoid the loss of important samples, we adopted k-means based on the Euclidean distance to cluster samples. New samples were generated though clustering, which had similar characteristics in the same cluster and were distinguished in the different clusters. The centroid of a cluster represents the overall characteristics of the whole cluster. In this way, important features are not discarded. Since the centroid of the cluster is calculated based on the average of the samples in the cluster, the centroid is not necessarily a real sample. So, we took the real samples with the smallest distance from the centroid.

### Processing Sparse Features

Sparse features means that the feature index is much larger than the actual number of nonzero features. In total, there were 891 different types of diagnosis in our data set. However, for a single patient, the number of diagnoses was quite few. This formed sparse-feature phenomena.

When sparse features occur, the sample is prone to having the problem of variable separation and multicollinearity. That is, a single variable or a linear combination of multiple variables can perfectly predict outcome events. However, this only works for small-size samples. It also leads to the situation in which the model gives an abnormally large weight to the variables and the results are unreliable [17,18,39]. Although there are many methods to optimize weights, such as gradient descent, a large number of zeros in features make the gradient tend to 0, and the parameters cannot be fully trained.

The processing of sparse features can be considered from both the model and the data themselves. From the point of view of the model, the parameter estimation bias of high-dimensional sparse data can be reduced through the optimization of the algorithm. For example, Firth regression [40] is used. The basic idea is to add a penalty term to the score function so as to reduce the deviation of the maximum-likelihood estimate of the parameter. This can solve the problem of variable separation and multicollinearity caused by sparse features to a large extent. From the point of view of the data themselves, it is necessary to transform the data to be processed into nonsparse data, and this transformation should retain the amount of information contained in the original data as much as possible. Considering the theme of our paper, our goal is to improve the quality of data rather than optimize the model algorithm. Therefore, we solved the problem of sparse features from the perspective of data. At present, there are many dimensionality reduction methods for high-dimensional sparse features, such as principal component analysis (PCA) [39], singular value decomposition (SVD) [41], and linear discriminant analysis (LDA) [42]. The essence of these methods is to map the original data to a low-dimensional space through a specific transformation form to solve the problem of data sparsity. Among these methods, LDA needs to reduce dimensionality based on sample labels. Considering that the actual data may not be able to carry labels, and the difference in label definitions will greatly affect the dimensionality reduction results, this supervised dimensionality reduction method is not conducive to being extended to other data scenarios [43]. Therefore, we considered using unsupervised dimensionality reduction methods, such as PCA, to transform our data.

PCA has been widely used in analysis with high-dimensional sparse features [44-46]. PCA essentially transforms the feature space of the original sample so that the new feature is a linear combination of the original features. The basic principle of principal component (PC) selection is to keep the maximum variance, and all PCs are orthogonal to one another. Thus, the phenomenon of multicollinearity is avoided. Therefore, new samples no longer have sparse features, which makes the ML model better fit the parameters.

In detail, new data can replace the original data as the input source for regression or classification models. Suppose 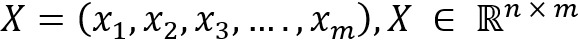 where each column represents a feature and each row is a sample. Assuming that the sample has been decentralized, 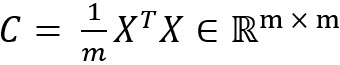 represents the covariance of matrix X. Let the transformed matrix Y = XV be D, which is derived as:



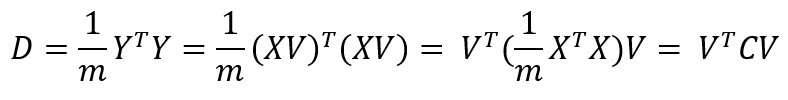



As C is a real symmetric matrix, according to the properties of the real symmetric matrix, its order m must have m unit orthogonal eigenvectors. That is, 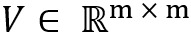 is a matrix that can make the original covariance matrix similar to diagonalization. Therefore, by solving m eigenvalues and eigenvectors of 
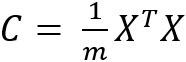
. By sorting the eigenvalues from large to small, we got λ = (λ_1_, λ_2_, …, λ_m_). There are the following relationships:



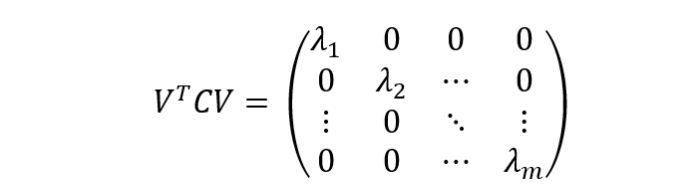



Take the first k columns of V as the basis for transforming m-dimensional features into k-dimensional features and record it as 
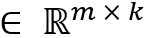
 the transformed sample matrix is Y = XP.

First, we manually merged similar diagnostic nouns according to prior knowledge, from 891 to 405. However, the data were obviously separated and sparse. For instance, none of the negative samples had a sudden cardiac arrest or sudden respiratory arrest diagnosis. Next, we only kept the diagnosis that appeared in more than 5% population. Finally, PCA was proposed for the remaining variables. The first 17 PCs that could explain 98.2% variance of the original sample were selected. Regression analysis was carried out on the samples after dimensionality reduction. The explanation of variables was achieved by counting the weight of the original variables on each PC.

### Ethical Considerations

After preliminary review, the project was found to be in line with relevant medical ethics requirements. If it is funded by the Hainan Major Science and Technology Program in 2020, the Hainan Medical Ethics Committee will perform its duties and strictly abide by relevant regulations and requirements for medical ethics and informed consent of patients to ensure ethical supervision and review during the implementation of the project (reference number: 00824482406).

## Results

### Data Preprocessing and Model Building

A comprehensive evaluation was carried out on the ED data set of the Hainan Hospital of Chinese PLA General Hospital. We developed a set of Python programs to implement our methods. Specifically, the program was developed in Microsoft Windows 10 (Intel (R) core (TM) i5-9500 CPU, 3GHz). All data preprocessing and model building were completed in Python (Python 3.8 Anaconda) using multiple Python data science libraries, mainly including Numpy, Pandas, Matplotlib, and Scikit-learn. In addition, codes on data interpolation, imbalance correction, and PC regression are currently available on GitHub [47].

### Data Collection and Cleaning

**Figure 2:**
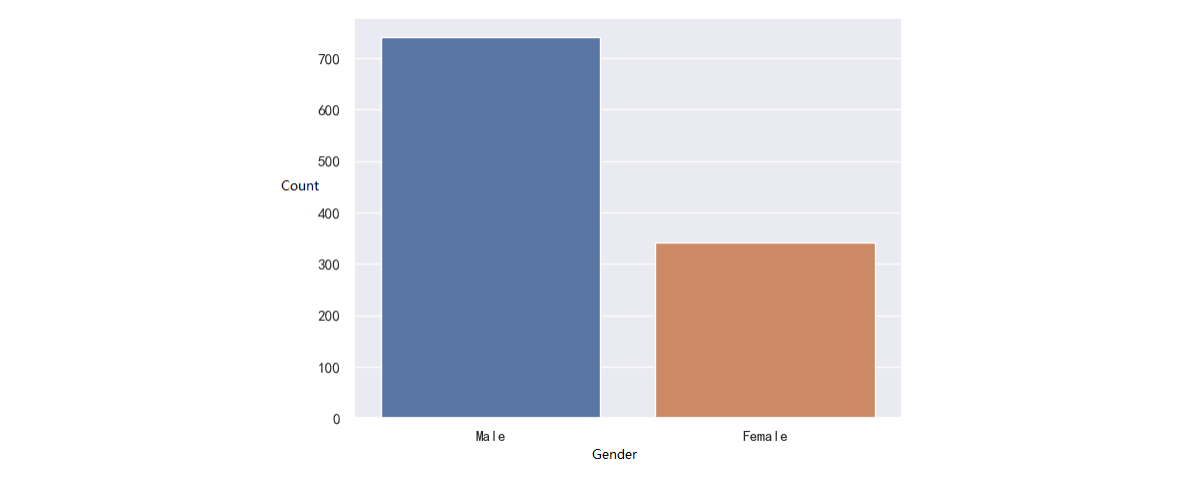
Distribution of the gender of patients with sudden death.

**Figure 3:**
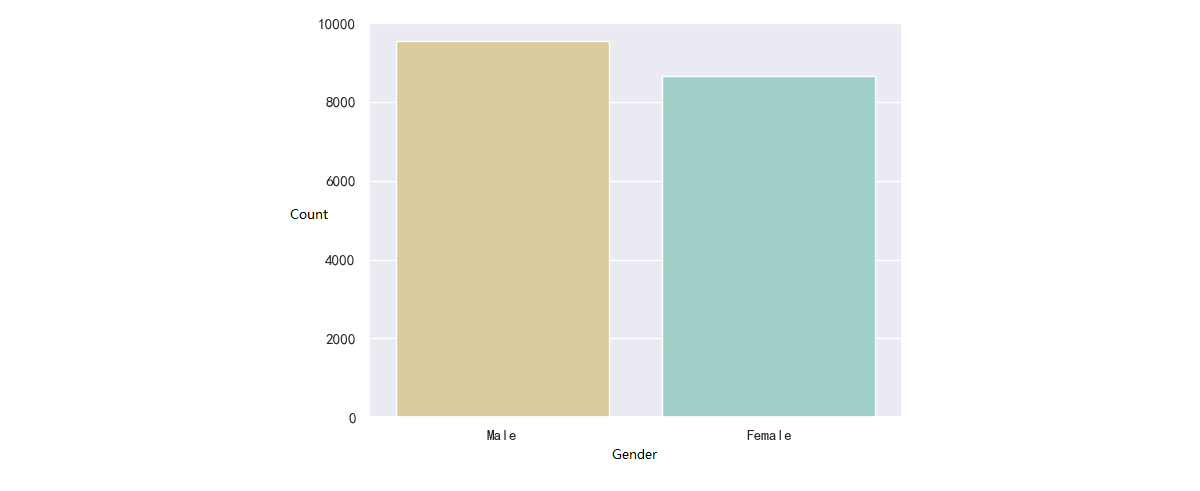
Distribution of the gender of patients without sudden death.

**Figure 4:**
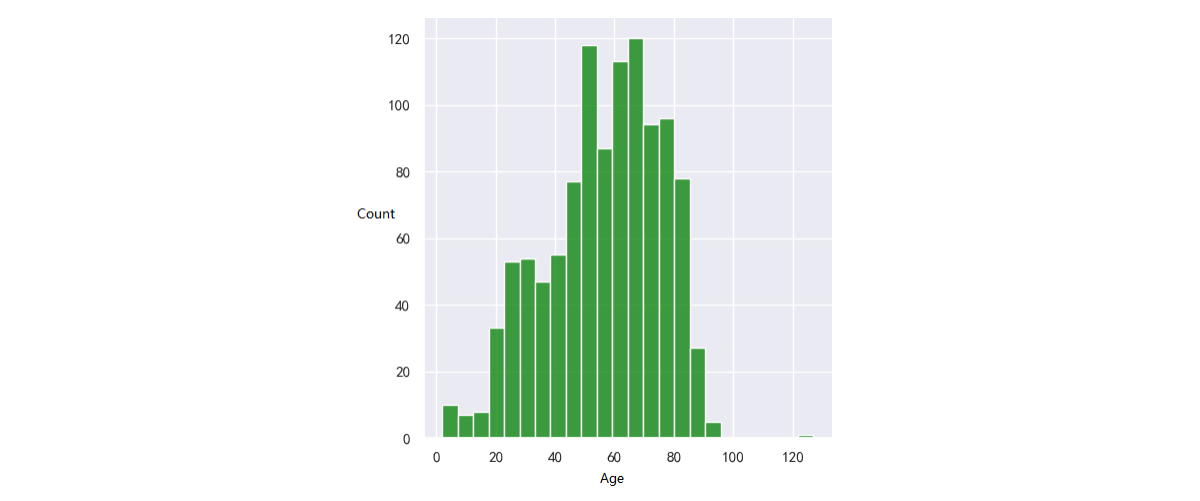
Distribution of age of patients with sudden death.

**Figure 5:**
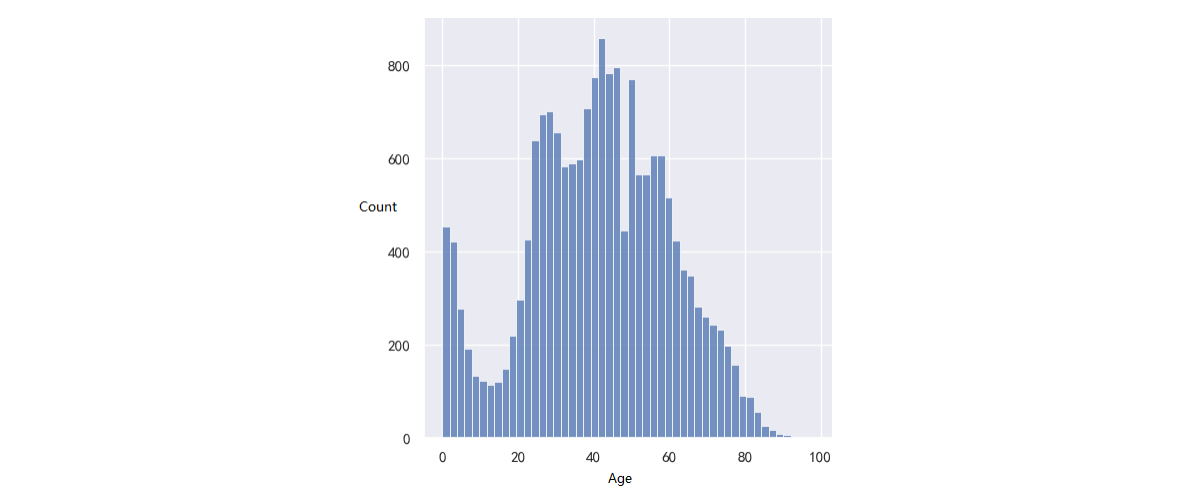
Distribution of patients of age with nonsudden death.

We collected the data of patients who went to the ED of the Hainan Hospital of Chinese PLA General Hospital from July 27, 2017, to May 6, 2021. In the sudden-death group, the data of 1085 patients were collected. In the non-sudden-death group, the data of 17,959 patients were collected. For the analysis of laboratory test data, we excluded patients who did not have any laboratory test records before sudden death. A total of 108 (10%) patients were excluded, and 977 (90%) patients with sudden death were used for the analysis of laboratory test data. For diagnostic data, we excluded patients who were missing diagnostic data from the visit. Finally, there were 1083 patients with sudden death and 615 patients with nonsudden death. We developed statistics on the baseline data of all patients, as shown in Supplementary Table S1 in Multimedia Appendix 1. Distributions of age and gender are visualized in Figures 2-5.

In the first group, there were 741 males (68.4%) and 342 females (31.6%), and 2 (0.2%) patients lacked gender information (Figure 2). The age varied between 45 and 80 years. The mean age was 56.4 years (SD 11.2). The quartile, median, and mode were 44, 59, and 68, respectively. In the second group, there were 9403 (52.4%) males and 8556 (47.6%) females. The age distribution is shown in Figures 4 and 5. The mean age was 41.6 years (SD 13.6). The quartile, median, and mode were 29, 42, and 48, respectively. For both groups, their distributions of age were akin to the normal distribution, which is consistent with a real-life situation.

### Variable Screening

To perform variable screening, that is, filtering out insignificant variables, we counted the total number of appearance and missing times. The second row of Table 2 shows the number of patients who had no corresponding data in the individual category. Moreover, we investigated the reasons missing data exist in all the 3 categories. For instance, there were 108 (10%) patients having no laboratory test. Among them, we could not find lab test data for 33 (30.6%) patients. For the remaining 75 (69.4%) patients, their lab tests appeared after the sudden-death event. There were 287 (26.4%) patients having no medication data. Sudden death had occurred before the medication was given, and the medication was in the doctor’s order record, such as an epinephrine injection, but was not recorded in the patient’s medication table.

There were 275 variables in the lab test category. For a given variable, not every patient (sample) had the value, namely a missing value. The missing ratio of a variable could be obtained by the number of cases having a missing value of that variable being divided by the total number of patients. The average ratio was 79.8%, as shown in the third row of Table 2. So, we set an 80% threshold to screen nonstatistically significant variables. Finally, 72 variables were kept in this category. These were patient age, gender, glucose, creatine kinase, inorganic phosphorus, total cholesterol, triglycerides, potassium, sodium, and calcium.

For diagnosis, 891 different types of diagnosis were obtained after the initial data collection. Because the diagnosis is recorded in the form of free text, 1 diagnosis item could have several different synonyms. By merging these texts into a unified name via manual review, we obtained 405 variables. The number of confirmed patients of each diagnostic variable was counted. Instead of an 80% threshold, 5% was considered. Considering both positive and negative samples, 18 diagnostic variables were kept. Among them, 11 (61.1%) variables were shared by both. These were myocardial infarction, chest distress, sudden cardiac arrest, fever, rib fracture, renal dysfunction, chest pain, diabetes, abdominal pain, pulmonary infection, respiratory arrest, trauma, atrial fibrillation, disturbance of consciousness, cerebral hemorrhage, cerebral infarction, coronary heart disease, and hypertension.

**Table 2:** Missing value ratios of variables of patients with sudden death.

	Laboratory tests (275 variables)	Medications (402 variables)	Diagnosis (891 variables)
Patients without data, n (%)	108 (10%)	287 (26.4%)	2 (0.18%)
Average ratio of missing values	79.8% (866/1085)	72.4% (786/1085)	99% (1080/1085)
Maximum ratio of missing values	90% (977/1085)	73.5% (797/1085)	100% (1085/1085)
Minimum ratio of missing values	25.8% (280/1085)	48.5% (526/1085)	58.4% (634/1085)

### Data Interpolation, Processing Imbalanced Data, and Sparse Features

In addition to age and gender, we used an RF to interpolate the missing values for each of the remaining variables. Nonmissing patient data were used as a training set to train the model to interpolate missing values. The training set was further split into training data (80%) and validation data (20%). The coefficient of determination R^2^ and the κ coefficient were used to test the consistency of the imputation results of continuous variables and categorical variables. In the interpolation process, the median of R^2^ was 0.623 (IQR 0.647) and the median of the κ coefficient was 0.444 (IQR 0.285).

Due to the extreme imbalance of our original data, the number of patients with sudden death only accounted for 5% (977/18,936) of the total sample size. We generated 4 different data ratios (1:10, 1:5, 1:2, and 1:1) through k-means to achieve undersampling. These data were used with the original ratio to evaluate models of different data ratios and then to verify the rationality of our sampling method.

### Validation by a Sudden-Death Case Study

#### Analyzing Risk Factors of Sudden Death

We constructed an LR model to analyze the patients’ laboratory test variables using a data set with a data ratio of 1:1 as the data source to filter variables. To reflect the degree of correlation between variables, continuous variables were treated as ordinal categorical variables. Taking the normal index range of the variables as a reference point, the test results of the patients were mapped into 3 categories: L (index is lower than the normal value), N (index is normal), and H (index is higher than the normal value). To determine the significant factors affecting the sudden death of patients and avoid a negative effect on the final analysis results, we first performed the chi-square test to filter out the variables and then excluded variables when *P*>.10. Next, LR univariate analysis was performed to filter out variables with *P*>.05. Tables 3 and 4, respectively, show the variables excluded by the chi-square test and the LR univariate analysis, and their *P* values. We reintroduced some of the excluded variables into the final candidate variable set according to the literature review and the advice of consulting medical experts, including urine specific gravity, chloride, hematocrit, sodium, magnesium, lactate dehydrogenase, urine ketone body test, red blood cell count, and serum albumin. These variables have no significant statistical significance but are clinically related to sudden death. Finally, we selected 4 subgroups from the set of variables with significant statistical significance. In addition, variables not statistically significant but related to outcome events were also grouped separately. The final 5 groups were subjected to LR multivariate analysis, and the groups were as follows:

- Group 1: qualitative test of creatinine, serum uric acid, urine protein
- Group 2: γ-glutamyl transferase, alanine aminotransferase, total bilirubin
- Group 3: international normalized ratio, platelet count, plasma prothrombin time
- Group 4: potassium, creatine kinase
- Group 5: urine specific gravity, chloride, hematocrit, sodium, magnesium, lactate dehydrogenase, urine ketone body test, red blood cell count, serum albumin

For each group, 500-fold bootstrapping was used for model training and evaluation [48]. Each bootstrap randomly split 70% of the data into the training set and 30% of the data into the test set. Finally, the mean values of AUROC, recall, and *F*_1_-score for 500 training sessions in each group were reported, and the AUROC also reported the 95% CI. Table 5 illustrates the model evaluation results of the 5 groups of variables. The performance parameters of group 2 were the best among the 5 groups of variables. In the recognition of patients with sudden death, a recall rate of 0.801 was obtained, the *F*_1_-score was 0.835, and the model’s AUROC was 0.843 (95% CI 0.842-0.844). The results showed that this set of variables can better identify patients with sudden death. Therefore, other group variables based on the group 2 variables were added successively, and AUROC was taken as the evaluation index. The added variables would be included in the final model if AUROC could be improved. In the end, 13 laboratory test risk variables related to sudden death events were determined, and the patient’s gender variable was retained as a demographic feature. In general, the final variables used included γ-glutamyl transferase, alanine aminotransferase, total bilirubin, creatinine, serum uric acid, the international standardized ratio, creatine kinase, the platelet count, potassium, sex, sodium, magnesium, chloride, and serum albumin. These variables were used to build the final LR model. Table 6 shows the results of LR multivariate analysis.

After determining the patient features for analysis, we split the original scale data into a training set (70%) and a test set (30%). For the training set, 4 different categories of data sets (1:1, 1:2, 1:5, 1:10) were formed by undersampling to train the model. Finally, the performance of the model was evaluated on the test set. The mean and 95% CI (500-fold bootstrapping) of the final AUROC, AUPRC, *F*_1_-score, and recall are shown in Supplementary Table S2 in Multimedia Appendix 1. In addition, we further used Brier scores to evaluate the calibration ability of models trained with different data ratios.

**Figure 6:**
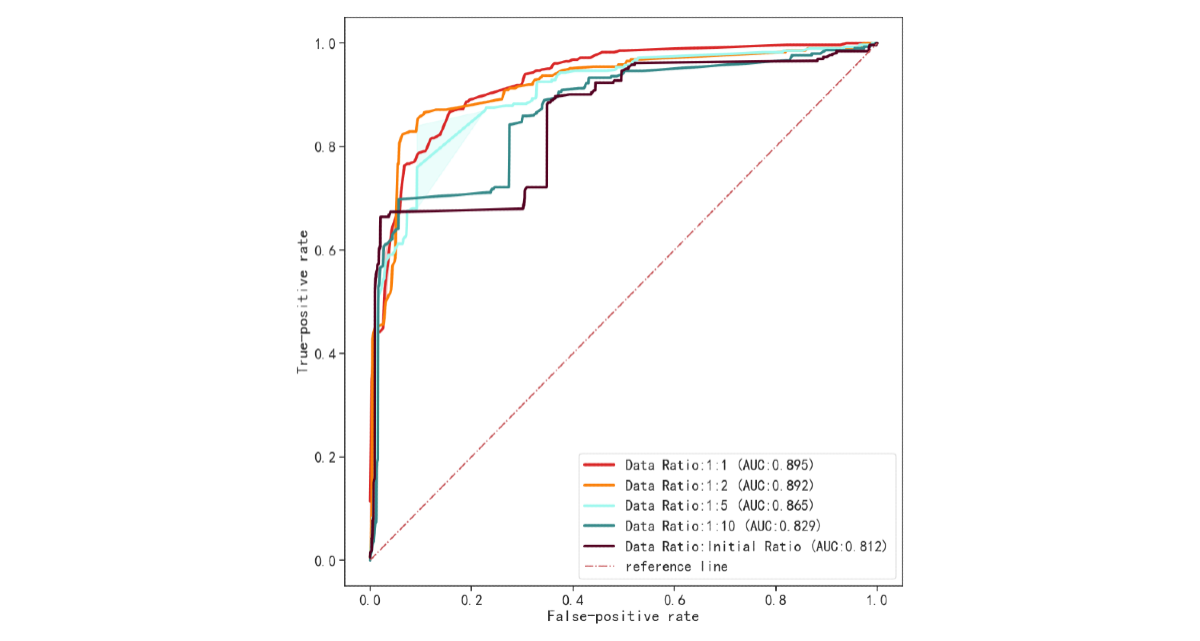
ROC curves of different data ratio. AUC: area under the curve; ROC: receiver operating characteristic.

**Figure 7:**
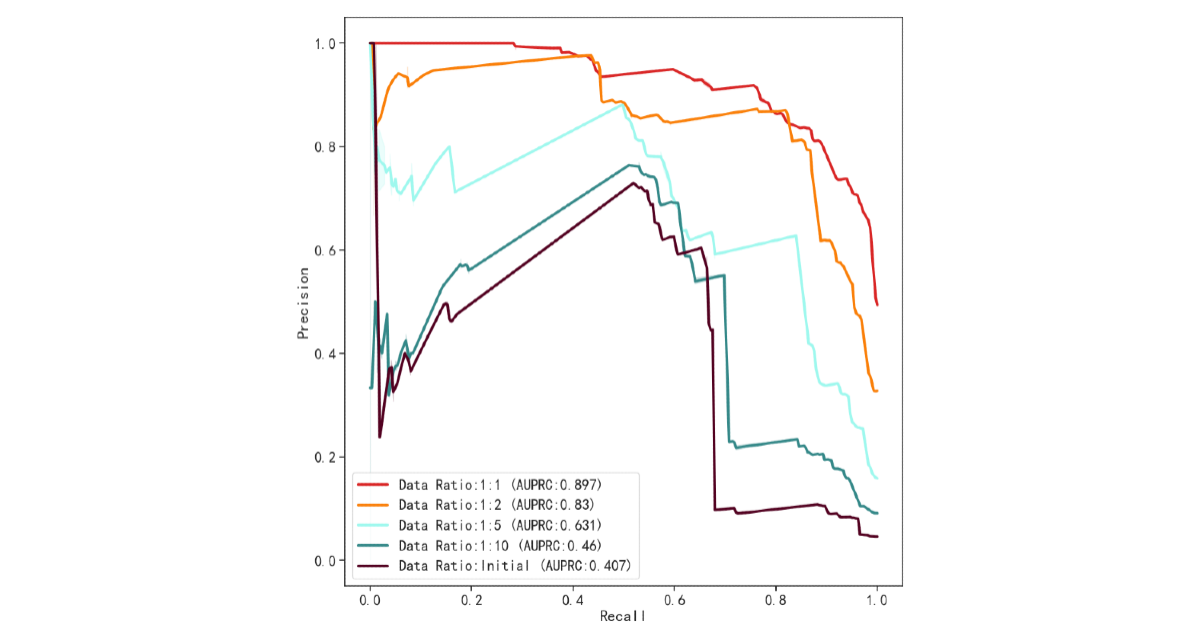
PR curves of different data ratio. AUPRC: area under the precision-recall curve; PR: precision-recall.

**Figure 8:**
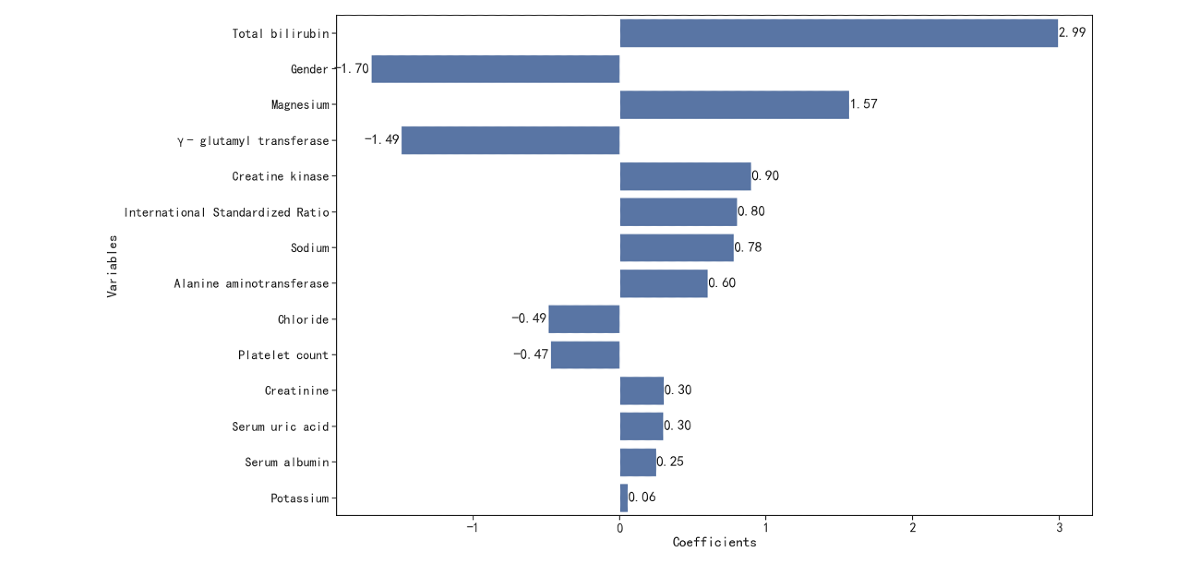
Visualization of logistic regression coefficients.

In general, as the data ratio tended to balance, the performance of the model gradually improved. Figures 6 and 7 show the model receiver operating characteristic (ROC) curve (Figure 6) and the precision-recall (PR) curve (Figure 7) of the 4 data ratios. In recognizing patients with sudden death, the best model obtained a recall rate of 0.863 (95% CI 0.862-0.865), the *F*_1_-score was 0.84 (95% CI 0.839-0.842), the AUROC of the model was 0.895 (95% CI 0.894-0.896), and the AUPRC was 0.897 (95% CI 0.896-0.899). The original scale data model performed the worst, with an AUROC of 0.812 (95% CI 0.811-0.813) and an AUPRC of 0.407 (95% CI 0.404-0.409). We plotted the reliability curves of 5 training sets with different data ratios on the same test set and calculated Brier scores (Supplementary Figure S1 in Multimedia Appendix 1). Consistent with the viewpoint mentioned by Geeven et al [49], imbalance correction actually weakened the clinical application value of the model, which was mainly manifested in the poor calibration ability of the model. With the increase in sampling, the calibration of the model was worse and the Brier score was 0.16 and 0.108 in the data ratio of 1:1 and the original data ratio, respectively. Imbalance correction can balance the sensitivity and specificity of the model to a greater extent and avoid biased errors in the model. Undersampling optimizes the AUROC, *F*_1_-score, and AUPRC of the model trained by the proportion of the original data. Although the Brier score in calibration improved, the gap was not large. To observe the risk factors of sudden death in patients more intuitively, we visualized the regression coefficients of the best model after performing LR(Figure 8) to observe the relationship between variables and sudden-death events.

**Table 3:** Statistics of variables filtered by the chi-square test.

Variable	*χ*^2^ (*df*)	*P* value
Monocytes	5.433 (6)	.49
Basophil	0.705 (4)	.95
Eosinophils	0.977 (4)	.91
Urine specific gravity determination	0 (2)	.99
Urine tube type	1.25 (4)	.87
Urine tube type (microscopic examination)	6.863 (8)	.98
Qualitative test of urinary bilirubin	13.185 (4)	.21
Mean erythrocyte hemoglobin concentration	7.828 (6)	.25
Chloride	4.649 (6)	.59
Erythrocyte volume distribution width measurement coefficient of variation (CV)	1.148 (4)	.89
Hematocrit assay	4.982 (6)	.55
Sodium	7.915 (6)	.24
Magnesium	10.22 (6)	.12

**Table 4:** Statistics of variables screened by LR^a^ univariate analysis.

Variable	Reference range	OR^b^ (95% CI)	*P* value
Lactate dehydrogenase	50.0-150.0 U/L	1.029 (0.94-1.127)	.53
Urine ketone body test	N/A^c^	0.912 (0.769-1.081)	.29
Red blood cell count	3.5-5.9 1012/L	0.827 (0.642-1.065)	.14
Serum albumin	35.0-50.0 g/L	0.893 (0.689-1.157)	.39
High-density lipoprotein cholesterol	1.0-1.6 mmol/L	0.961 (0.749-1.232)	.75

^a^LR: logistic regression.

^b^OR: odds ratio.

^c^N/A: not applicable.

**Table 5:** Comparing the performance of 5 groups of variables.

Group	Recall	*F*_1_-score	AUROC^a^ (95% CI)
1	0.478	0.6	0.683 (0.681-0.684)
2	0.801	0.835	0.843 (0.842-0.844)
3	0.606	0.687	0.725 (0.724-0.727)
4	0.484	0.605	0.686 (0.685-0.687)
5	0.852	0.651	0.562 (0.561-0.564)

^a^AUROC: area under the receiver operating characteristic curve.

**Table 6:** LR^a^ multivariate analysis.

Variable	Reference range	OR^b^ (95% CI)
γ-Glutamyl transferase	0.0-50.0 U/L	0.225 (0.222-0.228)
Alanine aminotransferase	5.0-40.0 U/L	1.828 (1.804-1.852)
Total bilirubin	0.0-21.0 μmol/L	19.954 (19.7-20.2)
Creatinine	30.0-110.0 μmol/L	1.352 (1.331-1.372)
Serum uric acid	104.0-444.0 μmol/L	1.346 (1.334-1.359)
International normalized ratio	0.8-1.2	2.23 (2.188-2.272)
Creatine kinase	24.0-320.0 U/L	2.457 (2.431-2.483)
Platelet count	100.0-300.0 ×10^9^/L	0.623 (0.617-0.629)
Potassium	3.5-5.1 mmol/L	1.057 (1.043-1.07)
Gender	Female	0.183 (0.182-0.184)
Sodium	135-145 mmol/L	2.182 (2.102-2.262)
Magnesium	0.8-1.0 mmol/L	4.807 (4.587-5.027)
Chloride	96.00-106.00 mmol/L	0.615 (0.603-0.627)
Serum albumin	35-51g/L	1.284 (1.268-1.3)

^a^LR: logistic regression.

^b^OR: odds ratio.

#### Development of Other ML Models

We use interpolated and undersampled data (data ratio 1:1) to train several other ML models and evaluate their performance. The training models included an RF [50], a gradient boosting machine (GBM) [51], a support vector machine (SVM) [52], and least absolute shrinkage and selection operator (LASSO) [53], which are also often used to develop medical prediction models [49,54]. We use 500-fold bootstrapping for internal validation. Each bootstrap used 70% data for training and the remaining 30% data for performance evaluation. The area under the curve (AUC), AUPRC, recall, and *F*_1_-score and their 95% CI values were reported. Before model training, a grid search was conducted to tune the best hyperparameter of each model through 5-fold cross-validation. The hyperparameter settings of each model are shown in Supplementary Table S7 in Multimedia Appendix 1. The ROC curve and PR curve of the models are shown in Supplementary Figures S2 and S3 in Multimedia Appendix 1, respectively, and the performance evaluation results are shown in Supplementary Table S8 in Multimedia Appendix 1. In general, the performance of the RF and GBM with an integrated scheme was the best, with an AUC of 0.936 (95% CI 0.934-0.937) and 0.931 (95% CI 0.93-0.932),respectively, and an *F*_1_-score of 0.857 (95% CI 0.856-0.858) and 0.821 (95% CI 0.82-0.823), respectively. This can benefit from the generalization and the ability to deal with complex feature relationships of the integrated model. The comprehensive decision results of multiple base learners are more stable than the single-model prediction results, and the performance is better. The SVM also performed better than the LR and LASSO, which are linear models, with an AUC of 0.913 (95% CI 0.912-0.914). This shows that there are some nonlinear features we used that made the linear model insufficient to recognize the relationship between these features.

#### Diagnostic Data Analysis Results

The final sample included 1083 patients with sudden death and 615 patients with nonsudden death. Table 7 shows the number of confirmed patients with 18 variables. The final diagnostic variables used included hypertension, myocardial infarction, cerebral hemorrhage, cardiac arrest, absolute pain, atmospheric fabric, fever, trauma, respiratory arrest, diabetes, corporate heart disease, and cerebral infarction.

We used 500-fold bootstrapping for internal validation of the model. For each bootstrap, 70% of the samples were randomly selected as the training set and 30% as the test set to evaluate the model. The final reported model performance was the mean and 95% CI of 500 results [48].

**Figure 9:**
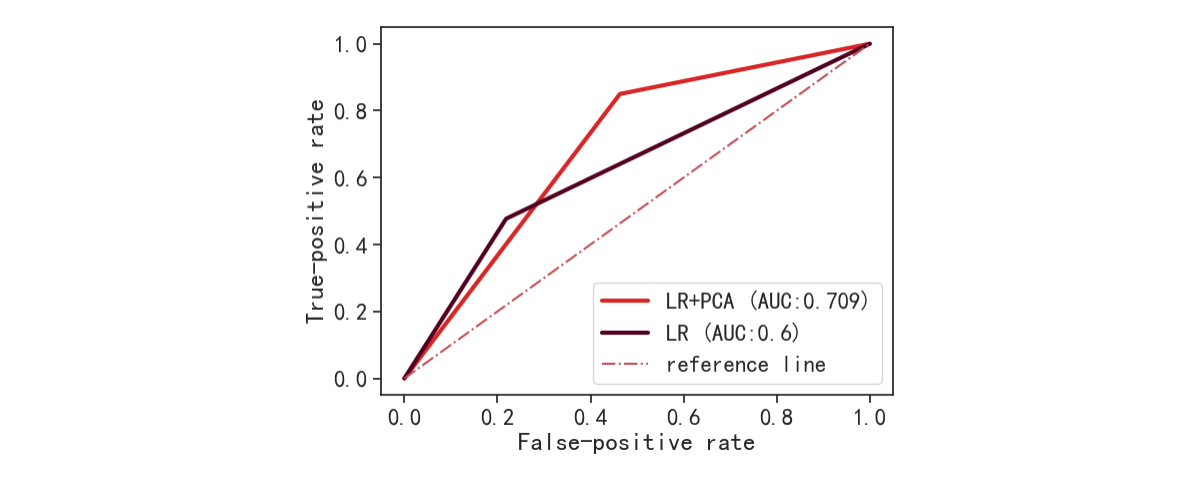
ROC curves of 2 models. AUC: area under the curve; LR: logistic regression; PCA: principal component analysis; ROC: receiver operating characteristic.

The first 17 PCs that could explain 98.2% of the variance of the original sample were selected as new variables for analysis. To observe the role of PCA, we compared the 2 schemes: the LR model using the original data and the LR model after dimensionality reduction using PCA. The LR model trained with the original data obtained a recall rate of 0.445 (95% CI 0.443-0.448), an *F*_1_-score of 0.562 95% CI 0.56-0.564), and an AUROC of 0.602 (95% CI 0.6-0.603). After PCA dimensionality reduction of the original data, the PC variable was used as the data source to train the LR model, and a recall rate of 0.746 (95% CI 0.731-0.76) was obtained, the *F*_1_-score was 0.73 (95% CI 0.721-0.738), and the AUROC of the model was 0.708 (95% CI 0.707-0.71). Figure 9 shows the ROC curves of the 2 models. The LR model using the original data had the phenomenon of variable separation, which is reflected in the abnormally high OR values of cardiac arrest and respiratory arrest (201568034532 and 1211118945) and an abnormal 95% CI, which makes the results unreliable. In addition, the performance of the model was poor, and only a recall rate of 0.445 was obtained in the identification of patients with sudden death, which means that the identification ability of the model for patients with sudden death is not strong. After PCA dimensionality reduction, the data were no longer sparse, the model parameters were better fitted, and the model performance improved to a certain extent. In addition, data conversion also eliminated the problems of variable separation and multicollinearity.

To determine the impact of various diagnostic variables on the sudden death of emergency patients, we statistically analyzed the results of multivariate analysis on 17 PCs input into the LR model. The OR of PC4, PC5, and PC6 was 3.044, 2.859, and 3.931, respectively, showing a significant correlation with sudden-death events (Table 8). In each PC, the magnitude of the loading, the elements in the PC, reflected the importance of the original variable in the PC (Supplementary Table S3 in Multimedia Appendix 1). The loadings of all components showed that cerebral infarction, hypertension, and pulmonary infection were the top 3 variables in PC4. In PC5 and PC6, the top 3 variables were consciousness disorder, diabetes, and fever. Based on the results of the 3 PCs, we believe that the 6 diagnoses of cerebral infarction, hypertension, pulmonary infection, consciousness disorder, diabetes, and fever are significantly associated with sudden death in emergency patients.

**Table 7:** Statistics of people diagnosed.

Variable	People with sudden death diagnosed, n (%)/people with nonsudden death diagnosed, n (%)
Myocardial infarction	57 (5.26)/23 (3.74)
Chest tightness	8 (0.74)/35 (5.69)
Cardiac arrest	120 (11.08)/0
Fever	50 (4.62)/43 (6.99)
Rib fracture	58 (5.36)/3 (0.49)
Abnormal renal function	42 (3.88)/35 (5.69)
Chest pain	18 (1.66)/38 (6.18)
Diabetes	65 (6.00)/66 (10.73)
Abdominal pain	30 (2.77)/45 (7.32)
Pulmonary infection	85 (7.85)/64 (10.41)
Respiratory arrest	106 (9.79)/0
Trauma	58 (5.36)/16 (2.60)
Atrial fibrillation	39 (3.60)/33 (5.37)
Disturbance of consciousness	82 (7.57)/17 (2.76)
Cerebral hemorrhage	77 (7.11)/26 (4.23)
Cerebral infarction	75 (6.93)/71 (11.54)
Coronary heart disease	29 (2.68)/39 (6.34)
Hypertension	65 (6.00)/106 (17.24)

**Table 8:** PC^a^ regression results

PC	OR^b^ (95% CI)
1	0.239 (0.235-0.242
2	2.429 (2.383-2.476)
3	1.19 (1.126-1.253)
4	3.044 (2.948-3.141)
5	2.859 (2.687-3.031)
6	3.931 (3.714-4.148)
7	1.49 (1.405-1.575)
8	1.699 (1.562-1.836)
9	2.104 (1.949-2.259)
10	2.153 (2.016-2.289)
11	2.451 (2.191-2.711)
12	2.031 (1.855-2.206)
13	1.457 (1.339-1.575)
14	0.949 (0.863-1.034)
15	1.423 (1.231-1.614)
16	2.546 (2.221-2.871)
17	0.182 (0.164-0.201)

^a^PC: principal component.

^b^OR: odds ratio.

## Discussion

### Principal Findings

In this paper, 3 ML schemes were proposed to deal with missing, imbalanced, and sparse features in the process of developing sudden-death prediction models using emergency medicine data, which improves the performance of the developed model. To solve the problem of missing data, we propose an RF method to use real data to interpolate missing data. In the interpolation process, the consistency of the interpolation results is checked by determining the coefficient R^2^ and the κ coefficient. From the interpolation results, the method shows the ability to correctly interpolate missing data. Imbalanced data are not conducive to obtaining accurate analysis results, and the model will be more inclined to predict new samples as patients with nonsudden death [15]. In view of this phenomenon, we used the k-means algorithm to generate multiple data sets with different proportions of different categories by undersampling to evaluate the model. The method based on k-means can better preserve the patient's characteristic information. This method will not lose some representative patient samples due to random discarding, thus reducing the bias caused by sampling. The results show that the comprehensive performance of the model gradually improves as the data tend to balance (Figures 3-5). However, imbalance correction will weaken the calibration ability of the model and increase the calibration error. Data sparsity is also not conducive to modeling and analysis. When the samples are too sparse, the results of the classifier based on maximum-likelihood estimation will become unreliable, because there may be variable separation and multicollinearity [18,55]. PC regression analysis is a method that uses PCA to extract the PC information about the original samples and uses PCs to replace the original variables for regression modeling [39]. In our diagnostic data, the LR model using the original data showed the phenomenon of variable separation, which led to unreliable results and poor performance. The performance of the PC regression model has been improved. In addition, we can analyze the diagnosis significantly related to the sudden death of emergency patients from the results of PC regression. These diagnoses are consistent with previous findings [9].

At present, there are many studies on the prediction of sudden death. Yu et al [54] constructed an ML model to predict sudden cardiac death (SCD) in 15,661 patients with atherosclerosis. The results showed that the ML model performs better than the standard Poisson regression model and the AUROC of the ML model was 0.89. Karen et al [56] trained an ML-based early warning model for identifying sudden infant death syndrome using the public data set “Lipidomic in sudden infant death syndrome.” The RF algorithm achieved an AUROC of 0.9 and a recall of 0.8. Ye et al [5] selected a variety of ML algorithms to build an early real-time early warning system (EWS) to predict the death risk of emergency patients and carried out prospective validation. The results showed that the EWS could give an early warning within 40 hours before sudden death, and the AUROC reached 0.884. Bhattacharya et al [57] used the electronic health records of 711 patients with hypertrophic myocardial cake and established an LR and naive Bayesian model with 22 variables, including statins, a family history of SCD, and left ventricular ejection fraction, to predict the risk of sudden death (ventricular fibrillation) in these patients. The sensitivity and specificity of the optimal model were 0.73 and 0.76, respectively, and the AUROC was 0.83. For our model, in the LR model constructed by using laboratory test data, the AUROC reached 0.895. After imbalance correction, the recall rate and AUPRC improved, reaching 0.863 and 0.897, respectively. Compared to the existing sudden-death prediction model based on ML, the performance of our model can achieve a similar effect, further indicating that our data-preprocessing methods can preserve the patient's characteristic information and improve the availability of emergency care.

### Limitations

This work also has some limitations. On the one hand, we only considered a single ML algorithm for data interpolation and did not discuss and compare the application of other possible ML algorithms in interpolation. It is possible that we overlooked the better performance of other methods. For example, for our data, due to the large proportion of missing and seriously imbalanced categorical variables, although we tried to adjust the relatively balanced data set to train the model, the κ coefficient improved to a certain extent but the effect was still poor. Therefore, a further discussion of ML methods that can handle a large number of missing and unbalanced categories or more reasonable feature processing may achieve better imputation results. Although imbalance correction can improve the sensitivity and specificity of the model, it can avoid biased errors of the model. However, this correction will also weaken the clinical application value of the model, lowering the calibration ability of the model and making it unable to accurately estimate the risk probability of patients. For the prediction model, the calibration ability of the model was not high, even on the original scale data set. Model calibration is another important characteristic of evaluating the clinical significance of prediction models. A well-calibrated model can provide more useful information for clinical decisions [58,59]. We can further consider using isotonic regression [60] to calibrate the model to improve its clinical application value. In addition, although the solution to deal with missing, imbalanced, and sparse features proposed by us is not the latest method, it is sufficient to solve the main data quality problems encountered in the development of prediction models for sudden death, which is reflected in the improvement of model performance and the consistency of the risk factors of sudden death obtained with the earlier literature results. In the future, we need to further explore the latest methods to solve these 3 data quality problems so as to extend the data-processing process to other data sets and provide a more reliable data source for prediction models. With regard to the construction of risk factor prediction models for patients with sudden death, we have a broad definition of sudden death, including patients who have undergone rescue or death events. These patients may include some nonemergency death cases, which may have a confusing effect on the final model. In addition, our feature selection was completely based on data, and only the remaining variables were trained in groups during the model training stage. This form can reduce the complexity of manually selecting features and also explore some potential risk variables. However, some clinically significant variables will also be discarded. Therefore, whether the model has clinical guiding significance remains to be further investigated. As a case study, we used LR as the main prediction model, which facilitated us to develop and analyze the risk factors of sudden death. However, the processing capacity of the LR model for nonlinear predictors is insufficient, resulting in insufficient performance of the developed model [17]. This can be seen from the results of other ML models we additionally developed (the RF and GBM had the best performance, with an AUC of 0.936 and 0.931, respectively, which are better than LR models). Therefore, in the future, we will further optimize the data-preprocessing process and try to develop ML models with better performance to improve the clinical usability.

### Conclusion

Our work proposes to use ML methods to deal with data quality issues, such as missing data, data imbalance, and sparse features in emergency data, so as to improve data availability. In addition, the risk factors of sudden death in emergency patients are obtained from our model analysis. As a preliminary analysis result, this result is also the basis for the later use of ML algorithms to build the feature selection and data analysis of the prediction model of sudden death in emergency patients.

## Appendix

Multimedia Appendix 1 Supplementary Tables and Figures.

## Abbreviations

AUC: area under the curve
AUPRC: area under the precision-recall curve
AUROC: area under the receiver operating characteristic curve
ED: emergency department
EMR: electronic medical record
EWS: early real-time early warning system
GBM: gradient boosting machine
LASSO: least absolute shrinkage and selection operator
LDA: linear discriminant analysis
MAR: missing at random deletion
MCAR: missing completely at random
ML: machine learning
MNAR: not missing at random
LR: logistic regression
OR: odds ratio
PC: principal component
PCA: principal component analysis
PR: precision-recall
RF: random forest
ROC: receiver operating characteristic
SCD: sudden cardiac death
SVM: support vector machine

## Data Availability

The data sets used and analyzed during this study are available from the first author upon reasonable request.
