# Supplementary material for: Dealing With Missing, Imbalanced, and Sparse Features During the Development of a Prediction Model for Sudden Death Using Emergency Medicine Data: Machine Learning Approach
Source: JMIR Med Inform. 2023 Jan 20;11:e38590. doi: 10.2196/38590 (PMC9898833; doi:10.2196/38590)

**Multimedia Appendix 1**

**Supplementary​ ​Table​ ​S1**.​ Baseline information of patients with sudden death and patients with no-sudden death.

| **Variables** | **Sudden Death(n=977)** | **Non-Sudden Death(n=17959)** | **Total(n=18936)** |
| --- | --- | --- | --- |
| Demographics | | | |
| Age,mean(SD) | 57.1(18.99) | 41.63(18.62) | 42.43(18.95) |
| Men,No.( %) | 663(67.86%) | 9403(52.36%) | 10213(53.23%) |
| Laboratory test | | | |
| Γ- glutamyl transferase, mean(SD) | 77.77(130.1) | 44.83(79.67) | 45.88(81.94) |
| Alanine aminotransferase, mean(SD) | 107.7(243.84) | 28.0(36.08) | 29.82(52.58) |
| Total bilirubin, mean(SD) | 19.95(49.14) | 12.72(16.6) | 12.91(18.26) |
| Creatinine, mean(SD) | 110.73(92.9) | 71.16(47.02) | 72.16(49.1) |
| Serum uric acid, mean(SD) | 404.78(149.48) | 331.8(102.31) | 333.83(104.59) |
| International normalized ratio, mean(SD) | 1.34(3.03) | 1.0(0.2) | 1.04(1.12) |
| Creatine kinase, mean(SD) | 851.81(8875.09) | 118.83(382.48) | 150.22(1877.37) |
| Platelet count, mean(SD) | 213.22(99.41) | 227.08(72.41) | 226.35(74.14) |
| Potassium, mean(SD) | 5.28(6.25) | 4.09(0.41) | 4.14(1.37) |
| Sodium, mean(SD) | 140.78(6.86) | 141.94(3.77) | 141.89(3.97) |
| Magnesium, mean(SD) | 0.97(0.31) | 0.85(0.11) | 0.86(0.12) |
| Chloride, mean(SD) | 101.13(6.74) | 104.09(3.46) | 103.96(3.72) |
| Serum albumin, mean(SD) | 38.48(7.29) | 45.2(4.7) | 45.02(4.91) |
| Diagnostic | | | |
| Miocardial infarction, No.( %) | 57(5.83%) | 23(0.13%) | 80(0.42%) |
| Chest tightness, No.( %) | 8(0.82%) | 35(0.19%) | 43(0.23%) |
| Cardiac arrest, No.( %) | 120(12.28%) | 0(0.0%) | 120(0.63%) |
| Fever, No.( %) | 50(5.12%) | 43(0.24%) | 93(0.49%) |
| Rib fracture, No.( %) | 58(5.94%) | 3(0.02%) | 61(0.32%) |
| Renal dysfunction, No.( %) | 42(4.3%) | 35(0.19%) | 77(0.41%) |
| Chest pain,No.( %) | 18(1.84%) | 38(0.21%) | 56(0.3%) |
| Diabetes, No.( %) | 65(6.65%) | 66(0.37%) | 131(0.69%) |
| Abdominal pain, No.( %) | 30(3.07%) | 45(0.25%) | 75(0.4%) |
| Pulmonary infection, No.( %) | 85(8.7%) | 64(0.36%) | 149(0.79%) |
| Respiratory arrest, No.( %) | 106(10.85%) | 0(0.0%) | 106(0.56%) |
| Trauma, No.( %) | 58(5.94%) | 16(0.09%) | 74(0.39%) |
| Atrial fibrillation, No.( %) | 39(3.99%) | 33(0.18%) | 72(0.38%) |
| Consciousness disorder, No.( %) | 82(8.39%) | 17(0.09%) | 99(0.52%) |
| Cerebral hemorrhage, No.( %) | 77(7.88%) | 26(0.14%) | 103(0.54%) |
| Cerebral infarction, No.( %) | 75(7.68%) | 71(0.4%) | 146(0.77%) |
| Coronary heart disease, No.( %) | 29(2.97%) | 39(0.22%) | 68(0.36%) |
| Hypertension, No.( %) | 65(6.65%) | 106(0.59%) | 171(0.9%) |

**Supplementary​ ​Table​ S2**.​ Performance of different category ratio.

|  | **AUROC** | **Recall** | **F1** | **AUPRC** |
| --- | --- | --- | --- | --- |
| **Ratio 1:1** | 0.895(0.894 - 0.896) | 0.857(0.856 - 0.859) | 0.839(0.838 - 0.84) | 0.898(0.897 - 0.9) |
| **Ratio 1:2** | 0.89(0.889 - 0.891) | 0.832(0.83 - 0.834) | 0.765(0.764 - 0.766) | 0.827(0.825 - 0.829) |
| **Ratio 1:5** | 0.857(0.856 - 0.859) | 0.665(0.659 - 0.671) | 0.629(0.626 - 0.633) | 0.62(0.617 - 0.622) |
| **Ratio 1:10** | 0.823(0.822 - 0.824) | 0.594(0.592 - 0.596) | 0.542(0.54 - 0.544) | 0.46(0.458 - 0.462) |
| **Ratio Initial** | 0.802(0.801 - 0.803) | 0.584(0.582 - 0.586) | 0.513(0.511 - 0.515) | 0.404(0.402 - 0.407) |

**Supplementary​ ​Table​ S3**.​ Loadings of all components (PC1 to PC5).

| **Variables** | **PC1** | **PC2** | **PC3** | **PC4** | **PC5** |
| --- | --- | --- | --- | --- | --- |
| Hypertension | 0.515 | 0.321 | -0.141 | -0.412 | -0.069 |
| Myocardial infarction | 0.006 | -0.046 | 0.03 | -0.101 | 0.044 |
| Cerebral hemorrhage | 0.233 | 0.168 | -0.213 | 0.289 | 0.011 |
| Cardiac arrest | -0.343 | 0.621 | 0.109 | 0.03 | 0.015 |
| Abdominal pain | 0.011 | -0.063 | 0.032 | -0.06 | -0.06 |
| Atrial fibrillation | 0.107 | 0.038 | 0.045 | 0.033 | -0.06 |
| Fever | 0.134 | -0.001 | 0.242 | 0.132 | -0.11 |
| Trauma | -0.035 | -0.065 | -0.049 | 0.009 | 0.057 |
| Respiratory arrest | -0.312 | 0.585 | 0.101 | 0.036 | 0.013 |
| Diabetes | 0.353 | 0.178 | 0.089 | -0.311 | 0.184 |
| Coronary heart disease | 0.168 | 0.073 | 0.045 | -0.094 | -0.021 |
| Cerebral infarction | 0.409 | 0.263 | -0.175 | 0.422 | -0.071 |
| Disturbance of consciousness | 0.094 | -0.013 | 0.013 | 0.192 | 0.327 |
| Chest tightness | 0.017 | -0.017 | 0.005 | -0.038 | -0.028 |
| Chest pain | 0.008 | -0.018 | 0.024 | -0.034 | -0.002 |
| Pulmonary infection | 0.273 | -0.02 | 0.64 | 0.333 | 0 |
| Rib fracture | 0.044 | 0.003 | -0.092 | 0.009 | 0.058 |
| Abnormal renal function | 0.118 | 0.052 | 0.079 | -0.012 | 0.013 |

**Supplementary​ ​Table​ S3**.​ Loadings of all components (PC6 to PC11)

| **Variables** | **PC6** | **PC7** | **PC8** | **PC9** | **PC10** | **PC11** |
| --- | --- | --- | --- | --- | --- | --- |
| Hypertension | -0.027 | -0.025 | -0.011 | -0.013 | -0.023 | -0.034 |
| Myocardial infarction | 0.023 | -0.003 | 0.102 | 0.091 | 0.097 | 0.194 |
| Cerebral hemorrhage | 0.041 | -0.057 | 0.019 | 0.017 | -0.032 | 0.156 |
| Cardiac arrest | 0.018 | 0.008 | 0.007 | 0.007 | 0.009 | 0.02 |
| Abdominal pain | -0.11 | 0.057 | 0.002 | -0.035 | -0.114 | 0.188 |
| Atrial fibrillation | -0.097 | 0.065 | 0.026 | 0.058 | 0.168 | 0.088 |
| Fever | -0.154 | 0.127 | 0.146 | 0.179 | 0.11 | -0.001 |
| Trauma | 0.115 | 0.002 | 0.007 | 0.101 | 0.27 | 0.144 |
| Respiratory arrest | 0.015 | 0.008 | 0.007 | 0.008 | 0.011 | 0.021 |
| Diabetes | 0.169 | 0.027 | 0.006 | 0.004 | -0.031 | -0.059 |
| Coronary heart disease | -0.046 | -0.011 | -0.004 | 0.017 | 0.09 | 0.099 |
| Cerebral infarction | -0.135 | 0.001 | -0.019 | -0.03 | -0.004 | -0.092 |
| Disturbance of consciousness | 0.445 | 0.179 | 0.048 | 0.021 | -0.017 | -0.013 |
| Chest tightness | -0.028 | -0.033 | -0.027 | -0.038 | -0.033 | -0.045 |
| Chest pain | -0.005 | -0.03 | -0.023 | -0.052 | -0.067 | -0.058 |
| Pulmonary infection | 0.035 | -0.096 | -0.059 | -0.071 | -0.054 | -0.01 |
| Rib fracture | 0.111 | -0.001 | 0.027 | 0.054 | 0.063 | 0.122 |
| Abnormal renal function | -0.006 | 0.08 | 0.011 | 0.04 | 0.133 | 0.238 |

**Supplementary​ ​Table​ S3**.​ Loadings of all components (PC12 to PC17)

| **Variables** | **PC12** | **PC13** | **PC14** | **PC15** | **PC16** | **PC17** |
| --- | --- | --- | --- | --- | --- | --- |
| Hypertension | -0.015 | -0.056 | -0.092 | -0.037 | -0.027 | -0.045 |
| Myocardial infarction | 0.182 | -0.04 | -0.09 | -0.019 | 0.001 | 0.069 |
| Cerebral hemorrhage | 0.119 | 0.092 | 0.048 | -0.043 | -0.152 | -0.023 |
| Cardiac arrest | 0.023 | 0.004 | 0.01 | 0.012 | 0.014 | 0.019 |
| Abdominal pain | 0.299 | 0.036 | -0.012 | 0.027 | 0.024 | 0.053 |
| Atrial fibrillation | 0.144 | 0.183 | 0.063 | 0.013 | 0.016 | 0.02 |
| Fever | -0.056 | 0.004 | 0.064 | 0.052 | 0.007 | 0.053 |
| Trauma | 0.178 | -0.047 | -0.087 | 0.011 | -0.063 | 0.042 |
| Respiratory arrest | 0.028 | 0.007 | 0.009 | 0.011 | 0.003 | 0.021 |
| Diabetes | -0.075 | 0.003 | -0.033 | -0.01 | 0.001 | 0.073 |
| Coronary heart disease | 0.12 | 0.124 | 0.361 | 0.074 | -0.037 | -0.223 |
| Cerebral infarction | -0.065 | -0.085 | -0.058 | 0.032 | 0.089 | 0.052 |
| Disturbance of consciousness | 0.012 | 0.011 | 0.041 | 0.01 | -0.036 | 0.012 |
| Chest tightness | -0.036 | 0.035 | 0.126 | 0.067 | -0.022 | 0.903 |
| Chest pain | -0.062 | 0.102 | 0.27 | 0.372 | 0.241 | -0.189 |
| Pulmonary infection | 0.015 | -0.021 | -0.054 | -0.029 | 0.005 | -0.018 |
| Rib fracture | 0.102 | 0.11 | 0.201 | 0.184 | 0.407 | 0.121 |
| Abnormal renal function | 0.141 | 0.004 | -0.016 | 0.018 | 0.025 | 0.06 |

**Supplementary​ ​Table​ S4**.​ Chi square analysis of all predictors (laboratory tests and demographics) related to sudden death

| **Variables** | **Odds Rate** | **95% CI** | **p value** |
| --- | --- | --- | --- |
| **Age, year** |  |  |  |
| 0~40 | Reference |  |  |
| 40~80 | 2.635 | 2.152 ~ 3.228 | <0.001 |
| >80 | 11.003 | 6.619 ~ 18.29 | <0.001 |
| **Gender** |  |  |  |
| Male | Reference |  |  |
| Female | 0.43 | 0.358 ~ 0.517 | <0.001 |
| **Γ- Glutamyltransferase, U/L** |  |  |  |
| 0.0~50.0 | Reference |  |  |
| 50.1~2043.0 | 2.848 | 2.363 ~ 3.433 | <0.001 |
| **Alanine Aminotransferase, U/L** |  |  |  |
| 5.0~40.0 | Reference |  |  |
| 0.0~5.0 | 1.444 | 0.289 ~ 7.215 | 0.421 |
| 40.1~10438.0 | 17.075 | 13.659 ~ 21.344 | <0.001 |
| **Neutrophils, Promotion** |  |  |  |
| 0.5~0.7 | Reference |  |  |
| 0.0~0.499 | 1.073 | 0.838 ~ 1.373 | <0.001 |
| 0.701~1.0 | 1.947 | 1.579 ~ 2.401 | <0.001 |
| **Lactate Dehydrogenase, U/L** |  |  |  |
| 50.0~150.0 | Reference |  |  |
| 150.1~16335.0 | 3.173 | 1.985 ~ 5.073 | <0.001 |
| **Low density lipoprotein cholesterol, mmol/L** |  |  |  |
| 2.0~3.4 | Reference |  |  |
| 0.0~1.99 | 0.02 | 0.005 ~ 0.081 | 0.313 |
| 3.41~1111.0 | 0.003 | 0.0 ~ 0.023 | 0.597 |
| **Monocyte, 10^9/L** |  |  |  |
| 0.03~0.37 | Reference |  |  |
| 0.0~0.029 | 1.501 | 0.954 ~ 2.36 | <0.001 |
| 0.081~2.0 | 0.858 | 0.684 ~ 1.075 | <0.001 |
| **Basophils, 10^9/L** |  |  |  |
| 0.0~0.01 | Reference |  |  |
| 0.011~0.46 | 0.736 | 0.358 ~ 1.51 | 0.012 |
| **Eosinophils, 10^9/L** |  |  |  |
| 0.0~0.5 | Reference |  |  |
| 0.51~2.0 | 0 | 0.0 ~ nan |  |
| **International normalized ratio** |  |  |  |
| 0.8~1.2 | Reference |  |  |
| 1.21~21.07 | 4.917 | 3.452 ~ 7.003 | <0.001 |
| **Aspartate Aminotransferase, U/L** |  |  |  |
| 0.0~40.0 | Reference |  |  |
| 40.1~15614.0 | 21.68 | 17.203 ~ 27.321 | <0.001 |
| **Small round epithelial cells, ul** |  |  |  |
| 0.0~3.0 | Reference |  |  |
| 3.1~1747.5 | 80.262 | 48.886 ~ 131.776 | <0.001 |
| **Urine epithelial cells,/ul** |  |  |  |
| 0.0~40.0 | Reference |  |  |
| 40.1~1948.3 | 0.023 | 0.009 ~ 0.063 | 0.093 |
| **Urine Specific Gravity** |  |  |  |
| 0.0~1.09 | Reference |  |  |
| **Urine tube type,/ul** |  |  |  |
| 0.0~1.5 | Reference |  |  |
| 1.51~336.74 | 0.844 | 0.626 ~ 1.137 | <0.001 |
| **Urine Crystals** |  |  |  |
| 0.0~15.0 | Reference |  |  |
| 15.1~5063.7 | 0.007 | 0.002 ~ 0.03 | 0.301 |
| **Urine ph determination** |  |  |  |
| 5.0~7.0 | Reference |  |  |
| 7.01~9.0 | 0.076 | 0.023 ~ 0.246 | 0.184 |
| **Urine white blood cells,/ul** |  |  |  |
| 0.0~30363.6 | Reference |  |  |
| 23.1~51497.5 | 18.722 | 12.122 ~ 28.917 | <0.001 |
| **Urea, mmol/L** |  |  |  |
| 1.8~7.5 | Reference |  |  |
| -0.95~1.79 | 0.193 | 0.043 ~ 0.873 | 0.368 |
| 7.51~119.66 | 1.45 | 1.103 ~ 1.906 | <0.001 |
| **Urine RBC,/ul** |  |  |  |
| 0.0~68322.0 | Reference |  |  |
| 20.4~90780.8 | 26.736 | 17.03 ~ 41.974 | <0.001 |
| **Urine bacterial count,/ul** |  |  |  |
| 0.0~400.0 | Reference |  |  |
| 400.1~93833.4 | 0.454 | 0.377 ~ 0.547 | <0.001 |
| **Mean corpuscular volume, fl** |  |  |  |
| 80.0~100.0 | Reference |  |  |
| 0.0~79.9 | 0.433 | 0.319 ~ 0.587 | <0.001 |
| 100.2~124.8 | 5.267 | 2.951 ~ 9.399 | 0.001 |
| **Mean corpuscular hemoglobin concentration, g/L** |  |  |  |
| 320.0~360.0 | Reference |  |  |
| 0.0~319.9 | 0.902 | 0.731 ~ 1.113 | <0.001 |
| 360.1~833.0 | 3.441 | 1.263 ~ 9.375 | 0.096 |
| **Mean corpuscular hemoglobin, pg** |  |  |  |
| 27.0~34.0 | Reference |  |  |
| 0.0~26.9 | 0.404 | 0.312 ~ 0.523 | <0.001 |
| 34.1~68.2 | 2.474 | 1.231 ~ 4.97 | 0.009 |
| **Mean platelet volume measurement, fl** |  |  |  |
| 6.0~11.5 | Reference |  |  |
| 11.6~15.0 | 1.945 | 1.439 ~ 2.629 | <0.001 |
| **Total cholesterol, mmol/L** |  |  |  |
| 3.1~5.7 | Reference |  |  |
| 5.71~17.52 | 0.002 | 0.0 ~ 0.014 | 0.583 |
| **Total bilirubin, umol/L** |  |  |  |
| 0.0~21.0 | Reference |  |  |
| 21.1~815.7 | 31.65 | 24.337 ~ 41.16 | <0.001 |
| **Total protein, g/L** |  |  |  |
| 60.0~80.0 | Reference |  |  |
| 0.0~60.1 | 0.746 | 0.511 ~ 1.089 | <0.001 |
| 80.1~113.2 | 0.321 | 0.205 ~ 0.501 | <0.001 |
| **Inorganic phosphorus, mmol/L** |  |  |  |
| 0.89~1.6 | Reference |  |  |
| -0.06~0.88 | 4.398 | 2.939 ~ 6.58 | <0.001 |
| 1.61~9.38 | 59.151 | 43.941 ~ 79.625 | <0.001 |
| **Chloride, mmol/L** |  |  |  |
| 94.0~110.0 | Reference |  |  |
| 0.0~93.9 | 1.713 | 1.013 ~ 2.897 | <0.001 |
| 110.1~156.4 | 0.76 | 0.347 ~ 1.664 | 0.023 |
| **Lymphocytes, 10^9/L** |  |  |  |
| 0.2~0.4 | Reference |  |  |
| 0.0~0.199 | 1.937 | 1.57 ~ 2.389 | <0.001 |
| 0.401~2.0 | 1.151 | 0.897 ~ 1.478 | <0.001 |
| **Triglyceride, mmol/L** |  |  |  |
| 0.4~1.7 | Reference |  |  |
| 1.71~1111.0 | 0.002 | 0.0 ~ 0.016 | 0.585 |
| **White blood cell count, 10^9/L** |  |  |  |
| 3.5~10.0 | Reference |  |  |
| 0.0~3.49 | 1.201 | 0.662 ~ 2.178 | 0.002 |
| 10.01~24194.0 | 2.827 | 2.3 ~ 3.475 | <0.001 |
| **Direct bilirubin, umol/L** |  |  |  |
| 0.0~7.0 | Reference |  |  |
| 7.01~700.9 | 27.814 | 21.563 ~ 35.876 | <0.001 |
| **Alkaline phosphatase, U/L** |  |  |  |
| 0.0~130.0 | Reference |  |  |
| 130.1~5135.0 | 0.347 | 0.247 ~ 0.488 | <0.001 |
| **Determination of red blood cell volume distribution width CV,%** |  |  |  |
| 0.0~14.5 | Reference |  |  |
| 14.6~36.0 | 0.88 | 0.697 ~ 1.112 | <0.001 |
| **Determination of hematocrit, L/L** |  |  |  |
| 0.35~0.52 | Reference |  |  |
| 0.0~0.349 | 0.87 | 0.698 ~ 1.085 | <0.001 |
| 0.521~39.1 | 2.757 | 0.874 ~ 8.696 | 0.168 |
| **Red blood cell count, 10^12/L** |  |  |  |
| 3.5~5.9 | Reference |  |  |
| 0.0~3.49 | 1.663 | 1.235 ~ 2.239 | <0.001 |
| 5.91~7.87 | 2.748 | 1.398 ~ 5.403 | 0.007 |
| **Creatinine, umol/L** |  |  |  |
| 30.0~110.0 | Reference |  |  |
| 0.0~29.9 | 0.065 | 0.016 ~ 0.273 | 0.326 |
| 110.1~2630.0 | 1.2 | 0.869 ~ 1.657 | <0.001 |
| **Creatine kinase, U/L** |  |  |  |
| 24~320.0 | Reference |  |  |
| 0.0~23.9 | 0.234 | 0.091 ~ 0.602 | 0.072 |
| 320.1~344000.0 | 7.529 | 5.85 ~ 9.689 | <0.001 |
| **Glucose, mmol/L** |  |  |  |
| 3.4~6.1 | Reference |  |  |
| 0.0~3.38 | 20.527 | 6.816 ~ 61.824 | 0.144 |
| 6.11~87.24 | 42.94 | 30.779 ~ 59.905 | <0.001 |
| **Platelet volume distribution width,%** |  |  |  |
| 9.0~17.0 | Reference |  |  |
| 17.0~25.6 | 2.041 | 1.301 ~ 3.2 | <0.001 |
| **Platelet specific volume measurement,%** |  |  |  |
| 0.0~0.23 | Reference |  |  |
| 0.24~0.4 | 0.674 | 0.559 ~ 0.813 | <0.001 |
| **Platelet count, 10^9/L** |  |  |  |
| 100.0~300.0 | Reference |  |  |
| 0.0~99.0 | 2.226 | 1.46 ~ 3.393 | <0.001 |
| 301.0~1308.0 | 0.571 | 0.436 ~ 0.749 | <0.001 |
| **Determination of plasma prothrombin time, s** |  |  |  |
| 13.0~16.0 | Reference |  |  |
| 0.0~12.9 | 6.789 | 5.335 ~ 8.639 | <0.001 |
| 16.1~250.0 | 2.814 | 1.878 ~ 4.215 | <0.001 |
| **Determination of plasma prothrombin activity,%** |  |  |  |
| 75.0~100.0 | Reference |  |  |
| 2.0~74.9 | 26.327 | 19.714 ~ 35.158 | <0.001 |
| 100.1~188.0 | 6.732 | 5.157 ~ 8.788 | <0.001 |
| **Determination of plasma activated partial prothrombin time, s** |  |  |  |
| 32.0~43.0 | Reference |  |  |
| 15.0~31.9 | 7.031 | 3.272 ~ 15.108 | 0.02 |
| 32.1~236.0 | 3.4 | 1.574 ~ 7.344 | 0.021 |
| **Determination of plasma fibrinogen, g/L** |  |  |  |
| 2.0~4.0 | Reference |  |  |
| 0.08~1.99 | 7.589 | 4.874 ~ 11.817 | <0.001 |
| 4.01~16.2 | 7.922 | 6.304 ~ 9.955 | <0.001 |
| **Serum uric acid, umol/L** |  |  |  |
| 104.0~444.0 | Reference |  |  |
| 0.0~103.4 | 0.181 | 0.023 ~ 1.435 | 0.627 |
| 444.1~1477.0 | 5.519 | 4.421 ~ 6.89 | <0.001 |
| **Serum albumin, g/L** |  |  |  |
| 35.0~50.0 | Reference |  |  |
| 0.0~34.9 | 0.702 | 0.514 ~ 0.957 | <0.001 |
| 50.1~61.5 | 0.106 | 0.042 ~ 0.267 | 0.065 |
| **Hemoglobin determination, g/L** |  |  |  |
| 120.0~175.0 | Reference |  |  |
| 0.0~119.9 | 0.685 | 0.558 ~ 0.84 | <0.001 |
| 175.1~225.0 | 9.463 | 2.204 ~ 40.634 | 0.34 |
| **Calcium, mmol/L** |  |  |  |
| 2.1~2.8 | Reference |  |  |
| 0.0~2.09 | 0.567 | 0.389 ~ 0.827 | <0.001 |
| 2.81~3.92 | 6.053 | 2.092 ~ 17.514 | 0.124 |
| **Sodium, mmol/L** |  |  |  |
| 130.0~150.0 | Reference |  |  |
| 0.0~129.9 | 0.759 | 0.347 ~ 1.661 | 0.023 |
| 150.1~227.3 | 3.105 | 1.314 ~ 7.338 | 0.043 |
| **Potassium, mmol/L** |  |  |  |
| 3.5~5.1 | Reference |  |  |
| 0.0~3.49 | 1.39 | 0.951 ~ 2.031 | <0.001 |
| 5.11~18.07 | 4.36 | 2.885 ~ 6.592 | <0.001 |
| **Magnesium, mmol/L** |  |  |  |
| 0.6~1.4 | Reference |  |  |
| 0.0~0.59 | 0.429 | 0.151 ~ 1.222 | 0.116 |
| 1.41~3.37 | 4.117 | 1.371 ~ 12.36 | 0.142 |
| **High density lipoprotein cholesterol, mmol/L** |  |  |  |
| 1.0~1.6 | Reference |  |  |
| 1.61~3.6 | 0.006 | 0.001 ~ 0.044 | 0.592 |

**Supplementary​ ​Table​ S5**.​ Univariate logistic regression analysis of risk factors for sudden death

| **Variables** | **Odds Rate** | **95% CI** | **p value** |
| --- | --- | --- | --- |
| **Age, year** |  |  |  |
| 0~40.0 | Reference |  |  |
| >80 | 5.1 | 3.158 ~ 8.236 | <0.001 |
| 40~80 | 0.819 | 0.732 ~ 0.916 | <0.001 |
| **Gender** |  |  |  |
| Male | Reference |  |  |
| Female | 0.977 | 0.895 ~ 1.067 | 0.605 |
| **Γ- Glutamyltransferase, U/L** |  |  |  |
| 0.0~50.0 | Reference |  |  |
| 50.1~2043.0 | 1.838 | 1.588 ~ 2.127 | <0.001 |
| **Alanine Aminotransferase, U/L** |  |  |  |
| 5.0~40.0 | Reference |  |  |
| 40.1~10438.0 | 3.94 | 3.375 ~ 4.6 | <0.001 |
| 0.0~5.0 | 3 | 0.606 ~ 14.864 | 0.178 |
| **Neutrophils, Promotion** |  |  |  |
| 0.5~0.7 | Reference |  |  |
| 0.701~1.0 | 1.569 | 1.32 ~ 1.864 | <0.001 |
| 0.0~0.499 | 1.157 | 0.932 ~ 1.436 | 0.187 |
| **Lactate Dehydrogenase, U/L** |  |  |  |
| 50.0~150.0 | Reference |  |  |
| 150.1~16335.0 | 1.029 | 0.94 ~ 1.127 | 0.533 |
| **Low density lipoprotein cholesterol, mmol/L** |  |  |  |
| 2.0~3.4 | Reference |  |  |
| 3.41~1111.0 | 0.004 | 0.001 ~ 0.032 | <0.001 |
| 0.0~1.99 | 36.492 | 8.957 ~ 148.664 | <0.001 |
| **Monocyte, 10^9/L** |  |  |  |
| 0.03~0.37 | Reference |  |  |
| 0.081~2.0 | 0.849 | 0.694 ~ 1.039 | 0.112 |
| 0.0~0.029 | 0.673 | 0.433 ~ 1.047 | 0.079 |
| **Basophils, 10^9/L** |  |  |  |
| 0.0~0.01 | Reference |  |  |
| 0.011~0.46 | 0.722 | 0.354 ~ 1.474 | 0.371 |
| **Eosinophils, 10^9/L** |  |  |  |
| 0.0~0.5 | Reference |  |  |
| 0.51~2.0 | 0.015 | 0.0 ~ 147346.844 | 0.61 |
| **International normalized ratio,** |  |  |  |
| 0.8~1.2 | Reference |  |  |
| 1.21~21.07 | 4.146 | 2.948 ~ 5.831 | <0.001 |
| **Aspartate Aminotransferase, U/L** |  |  |  |
| 0.0~40.0 | Reference |  |  |
| 40.1~15614.0 | 4.389 | 3.741 ~ 5.149 | <0.001 |
| **Small round epithelial cells, ul** |  |  |  |
| 0.0~3.0 | Reference |  |  |
| 3.1~1747.5 | 2.324 | 2.071 ~ 2.609 | <0.001 |
| **Urine epithelial cells,/ul** |  |  |  |
| 0.0~40.0 | Reference |  |  |
| 40.1~1948.3 | 0.026 | 0.01 ~ 0.072 | <0.001 |
| **Urine Specific Gravity** |  |  |  |
| 0.0~1.09 | Reference |  |  |
| **Urine tube type,/ul** |  |  |  |
| 0.0~1.5 | Reference |  |  |
| 1.51~336.74 | 0.838 | 0.631 ~ 1.113 | 0.222 |
| **Urine Crystals** |  |  |  |
| 0.0~15.0 | Reference |  |  |
| 15.1~5063.7 | 0.009 | 0.002 ~ 0.037 | <0.001 |
| **Urine ph determination** |  |  |  |
| 5.0~7.0 | Reference |  |  |
| 7.01~9.0 | 0.077 | 0.024 ~ 0.249 | <0.001 |
| 0.0~4.99 | 65.289 | 0.0 ~ 628084630.375 | 0.61 |
| **Urine white blood cells,/ul** |  |  |  |
| 0.0~30363.6 | Reference |  |  |
| 23.1~51497.5 | 1.385 | 1.255 ~ 1.527 | <0.001 |
| **Urea, mmol/L** |  |  |  |
| 1.8~7.5 | Reference |  |  |
| 7.51~119.66 | 1.366 | 1.057 ~ 1.766 | 0.017 |
| -0.95~1.79 | 5.498 | 1.219 ~ 24.802 | 0.027 |
| **Urine RBC,/ul** |  |  |  |
| 0.0~68322.0 | Reference |  |  |
| 20.4~90780.8 | 1.517 | 1.372 ~ 1.678 | <0.001 |
| **Urine bacterial count,/ul** |  |  |  |
| 0.0~400.0 | Reference |  |  |
| 400.1~93833.4 | 0.6 | 0.519 ~ 0.695 | <0.001 |
| **Mean corpuscular volume, fl** |  |  |  |
| 80.0~100.0 | Reference |  |  |
| 100.2~124.8 | 5.286 | 2.986 ~ 9.358 | <0.001 |
| 0.0~79.9 | 2.303 | 1.725 ~ 3.075 | <0.001 |
| **Mean corpuscular hemoglobin concentration, g/L** |  |  |  |
| 320.0~360.0 | Reference |  |  |
| 360.1~833.0 | 3.4 | 1.254 ~ 9.216 | 0.016 |
| 0.0~319.9 | 1.122 | 0.933 ~ 1.35 | 0.222 |
| **Mean corpuscular hemoglobin, pg** |  |  |  |
| 27.0~34.0 | Reference |  |  |
| 34.1~68.2 | 2.727 | 1.367 ~ 5.442 | 0.004 |
| 0.0~26.9 | 2.247 | 1.769 ~ 2.855 | <0.001 |
| **Mean platelet volume measurement, fl** |  |  |  |
| 6.0~11.5 | Reference |  |  |
| 11.6~15.0 | 1.781 | 1.337 ~ 2.372 | <0.001 |
| 0.0~5.9 | 180.213 | 0.0 ~ 24021170635.106 | 0.586 |
| **Total cholesterol, mmol/L** |  |  |  |
| 3.1~5.7 | Reference |  |  |
| 5.71~17.52 | 0.003 | 0.0 ~ 0.022 | <0.001 |
| 0.0~3.09 | 3649.923 | 0.0 ~ 28956111066680.17 | 0.481 |
| **Total bilirubin, umol/L** |  |  |  |
| 0.0~21.0 | Reference |  |  |
| 21.1~815.7 | 8.364 | 6.705 ~ 10.433 | <0.001 |
| **Total protein, g/L** |  |  |  |
| 60.0~80.0 | Reference |  |  |
| 80.1~113.2 | 0.338 | 0.218 ~ 0.522 | <0.001 |
| 0.0~60.1 | 1.275 | 0.883 ~ 1.839 | 0.195 |
| **Inorganic phosphorus, mmol/L** |  |  |  |
| 0.89~1.6 | Reference |  |  |
| 1.61~9.38 | 10.929 | 8.556 ~ 13.959 | <0.001 |
| -0.06~0.88 | 1.231 | 0.854 ~ 1.775 | 0.266 |
| **Chloride, mmol/L** |  |  |  |
| 94.0~110.0 | Reference |  |  |
| 110.1~156.4 | 0.733 | 0.337 ~ 1.597 | 0.435 |
| 0.0~93.9 | 0.605 | 0.361 ~ 1.016 | 0.057 |
| **Lymphocytes, 10^9/L** |  |  |  |
| 0.2~0.4 | Reference |  |  |
| 0.401~2.0 | 0.921 | 0.739 ~ 1.148 | 0.465 |
| 0.0~0.199 | 0.645 | 0.542 ~ 0.768 | <0.001 |
| **Triglyceride, mmol/L** |  |  |  |
| 0.4~1.7 | Reference |  |  |
| 1.71~1111.0 | 0.003 | 0.0 ~ 0.023 | <0.001 |
| 0.0~0.39 | 492.594 | 0.0 ~ 7038440568.881 | 0.461 |
| **White blood cell count, 10^9/L** |  |  |  |
| 3.5~10.0 | Reference |  |  |
| 10.01~24194.0 | 2.06 | 1.727 ~ 2.456 | <0.001 |
| 0.0~3.49 | 1.143 | 0.636 ~ 2.053 | 0.655 |
| **Direct bilirubin, umol/L** |  |  |  |
| 0.0~7.0 | Reference |  |  |
| 7.01~700.9 | 7.546 | 6.106 ~ 9.326 | <0.001 |
| **Alkaline phosphatase, U/L** |  |  |  |
| 0.0~130.0 | Reference |  |  |
| 130.1~5135.0 | 0.371 | 0.267 ~ 0.515 | <0.001 |
| **Determination of red blood cell volume distribution width CV,%** |  |  |  |
| 0.0~14.5 | Reference |  |  |
| 14.6~36.0 | 0.879 | 0.711 ~ 1.087 | 0.235 |
| **Determination of hematocrit, L/L** |  |  |  |
| 0.35~0.52 | Reference |  |  |
| 0.521~39.1 | 2.75 | 0.876 ~ 8.636 | 0.083 |
| 0.0~0.349 | 1.152 | 0.946 ~ 1.404 | 0.16 |
| **Red blood cell count, 10^12/L** |  |  |  |
| 3.5~5.9 | Reference |  |  |
| 5.91~7.87 | 2.5 | 1.28 ~ 4.883 | 0.007 |
| 0.0~3.49 | 0.661 | 0.498 ~ 0.877 | 0.004 |
| **Creatinine, umol/L** |  |  |  |
| 30.0~110.0 | Reference |  |  |
| 110.1~2630.0 | 1.189 | 0.873 ~ 1.62 | 0.272 |
| 0.0~29.9 | 15.492 | 3.709 ~ 64.711 | <0.001 |
| Creatine kinase, U/L |  |  |  |
| 24~320.0 | Reference |  |  |
| 320.1~344000.0 | 4.73 | 3.763 ~ 5.946 | <0.001 |
| 0.0~23.9 | 6.8 | 2.66 ~ 17.386 | <0.001 |
| **Glucose, mmol/L** |  |  |  |
| 3.4~6.1 | Reference |  |  |
| 6.11~87.24 | 2.789 | 2.46 ~ 3.162 | <0.001 |
| 0.0~3.38 | 0.75 | 0.26 ~ 2.162 | 0.594 |
| **Platelet volume distribution width,%** |  |  |  |
| 9.0~17.0 | Reference |  |  |
| 17.0~25.6 | 1.933 | 1.244 ~ 3.004 | 0.003 |
| **Platelet specific volume measurement,%** |  |  |  |
| 0.0~0.23 | Reference |  |  |
| 0.24~0.4 | 0.753 | 0.646 ~ 0.877 | <0.001 |
| **Platelet count, 10^9/L** |  |  |  |
| 100.0~300.0 | Reference |  |  |
| 301.0~1308.0 | 0.576 | 0.447 ~ 0.741 | <0.001 |
| 0.0~99.0 | 0.446 | 0.296 ~ 0.672 | <0.001 |
| **Determination of plasma prothrombin time, s** |  |  |  |
| 13.0~16.0 | Reference |  |  |
| 16.1~250.0 | 1.634 | 1.108 ~ 2.41 | 0.013 |
| 0.0~12.9 | 0.254 | 0.205 ~ 0.314 | <0.001 |
| **Determination of plasma prothrombin activity,%** |  |  |  |
| 75.0~100.0 | Reference |  |  |
| 100.1~188.0 | 1.907 | 1.526 ~ 2.383 | <0.001 |
| 2.0~74.9 | 0.134 | 0.104 ~ 0.172 | <0.001 |
| **Determination of plasma activated partial prothrombin time, s** |  |  |  |
| 32.0~43.0 | Reference |  |  |
| 32.1~236.0 | 0.648 | 0.559 ~ 0.75 | <0.001 |
| 15.0~31.9 | 0.747 | 0.665 ~ 0.838 | <0.001 |
| **Determination of plasma fibrinogen, g/L** |  |  |  |
| 2.0~4.0 | Reference |  |  |
| 4.01~16.2 | 3.75 | 3.086 ~ 4.557 | <0.001 |
| 0.08~1.99 | 0.278 | 0.182 ~ 0.426 | <0.001 |
| **Serum uric acid, umol/L** |  |  |  |
| 104.0~444.0 | Reference |  |  |
| 444.1~1477.0 | 3.383 | 2.788 ~ 4.106 | <0.001 |
| 0.0~103.4 | 9 | 1.14 ~ 71.036 | 0.037 |
| **Serum albumin, g/L** |  |  |  |
| 35.0~50.0 | Reference |  |  |
| 50.1~61.5 | 0.111 | 0.044 ~ 0.28 | <0.001 |
| 0.0~34.9 | 1.355 | 1.008 ~ 1.823 | 0.044 |
| **Hemoglobin determination, g/L** |  |  |  |
| 120.0~175.0 | Reference |  |  |
| 175.1~225.0 | 10 | 2.337 ~ 42.781 | 0.002 |
| 0.0~119.9 | 1.382 | 1.158 ~ 1.649 | <0.001 |
| **Calcium, mmol/L** |  |  |  |
| 2.1~2.8 | Reference |  |  |
| 2.81~3.92 | 6 | 2.082 ~ 17.292 | 0.001 |
| 0.0~2.09 | 1.778 | 1.234 ~ 2.561 | 0.002 |
| **Sodium, mmol/L** |  |  |  |
| 130.0~150.0 | Reference |  |  |
| 150.1~227.3 | 3 | 1.275 ~ 7.057 | 0.012 |
| 0.0~129.9 | 1.364 | 0.626 ~ 2.969 | 0.435 |
| **Potassium, mmol/L** |  |  |  |
| 3.5~5.1 | Reference |  |  |
| 5.11~18.07 | 3.8 | 2.542 ~ 5.681 | <0.001 |
| 0.0~3.49 | 0.825 | 0.572 ~ 1.192 | 0.306 |
| **Magnesium, mmol/L** |  |  |  |
| 0.6~1.4 | Reference |  |  |
| 1.41~3.37 | 4 | 1.337 ~ 11.964 | 0.013 |
| 0.0~0.59 | 2.4 | 0.846 ~ 6.812 | 0.1 |
| **High density lipoprotein cholesterol, mmol/L** |  |  |  |
| 1.0~1.6 | Reference |  |  |
| 1.61~3.6 | 0.008 | 0.001 ~ 0.056 | <0.001 |
| 0.0~0.99 | 9924.238 | 0.0 ~ 508511985458.633 | 0.31 |

**Supplementary​ ​Table​ S6**.​ Multivariate logistic regression analysis of risk factors for sudden death

| Variables | Odds Rate | 95% CI | p value |
| --- | --- | --- | --- |
| **Gender** |  |  |  |
| Male | Reference |  |  |
| Female | 0.122 | 0.098 ~ 0.152 | <0.001 |
| **Γ- Glutamyltransferase, U/L** |  |  |  |
| 0.0~50.0 | Reference |  |  |
| 50.1~2043.0 | 0.46 | 0.326 ~ 0.65 | <0.001 |
| **Alanine Aminotransferase, U/L** |  |  |  |
| 5.0~40.0 | Reference |  |  |
| 40.1~10438.0 | 3.178 | 2.184 ~ 4.624 | <0.001 |
| 0.0~5.0 | 0.601 | 0.099 ~ 3.635 | 0.579 |
| **Total bilirubin, umol/L** |  |  |  |
| 0.0~21.0 | Reference |  |  |
| 21.1~815.7 | 15.857 | 11.069 ~ 22.715 | <0.001 |
| **Creatinine, umol/L** |  |  |  |
| 30.0~110.0 | Reference |  |  |
| 110.1~2630.0 | 1.33 | 0.81 ~ 2.183 | 0.259 |
| 0.0~29.9 | 12.734 | 2.418 ~ 67.067 | 0.003 |
| Serum uric acid, umol/L |  |  |  |
| 104.0~444.0 | Reference |  |  |
| 444.1~1477.0 | 2.906 | 2.1 ~ 4.023 | <0.001 |
| 0.0~103.4 | 5.015 | 0.334 ~ 75.376 | 0.244 |
| **International normalized ratio** |  |  |  |
| 0.8~1.2 | Reference |  |  |
| 1.21~21.07 | 3.914 | 2.354 ~ 6.509 | <0.001 |
| **Creatine kinase, U/L** |  |  |  |
| 24~320.0 | Reference |  |  |
| 320.1~344000.0 | 3.488 | 2.494 ~ 4.877 | <0.001 |
| 0.0~23.9 | 4.788 | 1.339 ~ 17.127 | 0.016 |
| **Platelet count, 10^9/L** |  |  |  |
| 100.0~300.0 | Reference |  |  |
| 301.0~1308.0 | 0.833 | 0.554 ~ 1.253 | 0.381 |
| 0.0~99.0 | 1.982 | 1.004 ~ 3.911 | 0.049 |
| **Potassium, mmol/L** |  |  |  |
| 3.5~5.1 | Reference |  |  |
| 5.11~18.07 | 4.479 | 2.44 ~ 8.22 | <0.001 |
| 0.0~3.49 | 0.184 | 0.113 ~ 0.299 | <0.001 |
| **Sodium, mmol/L** |  |  |  |
| 130.0~150.0 | Reference |  |  |
| 150.1~227.3 | 3.287 | 1.071 ~ 10.095 | 0.038 |
| 0.0~129.9 | 5.018 | 1.361 ~ 18.498 | 0.015 |
| **Magnesium, mmol/L** |  |  |  |
| 0.6~1.4 | Reference |  |  |
| 1.41~3.37 | 1.804 | 0.506 ~ 6.437 | 0.363 |
| 0.0~0.59 | 5.729 | 1.414 ~ 23.205 | 0.014 |
| **Chloride, mmol/L** |  |  |  |
| 94.0~110.0 | Reference |  |  |
| 110.1~156.4 | 1.048 | 0.351 ~ 3.131 | 0.933 |
| 0.0~93.9 | 0.234 | 0.106 ~ 0.516 | <0.001 |
| **Serum albumin, g/L** |  |  |  |
| 35.0~50.0 | Reference |  |  |
| 50.1~61.5 | 0.185 | 0.055 ~ 0.62 | 0.006 |
| 0.0~34.9 | 2.052 | 1.227 ~ 3.43 | 0.006 |

**Supplementary​ ​Table​ ​S7**.​ Hyperparameter setting of four machine learning models.

| Model | Hyperparameter |
| --- | --- |
| SVM | C=1.5 |
|  | kernel='rbf' |
|  | gamma=0.05 |
| RF | max_depth=6 |
|  | n_estimators=200 |
|  | min_samples_split=4 |
| LASSO | max_iter=2300 |
|  | alpha=0.01 |
| GBM | max_depth=3 |
|  | n_estimators=220 |
|  | min_samples_split=4 |

**Supplementary​ ​Table​ S8**.​ Performance of five machine learning models.

| Model | AUC | RECALL | F1 | AUPRC |
| --- | --- | --- | --- | --- |
| SVM | 0.913(0.912 - 0.914) | 0.809(0.807 - 0.811) | 0.845(0.844 - 0.846) | 0.81(0.808 - 0.811) |
| RF | 0.936(0.934 - 0.937) | 0.825(0.823 - 0.827) | 0.857(0.856 - 0.858) | 0.822(0.821 - 0.824) |
| LASSO | 0.846(0.845 - 0.847) | 0.788(0.786 - 0.79) | 0.836(0.834 - 0.837) | 0.806(0.804 - 0.807) |
| GBM | 0.931(0.93 - 0.932) | 0.85(0.848 - 0.851) | 0.864(0.863 - 0.865) | 0.821(0.82 - 0.823) |
| LR | 0.91(0.909 - 0.911) | 0.863(0.862 - 0.865) | 0.84(0.839 - 0.842) | 0.809(0.807 - 0.811) |

**Supplementary​ ​Figure S1**.​ Reliability curves of five data ratio.


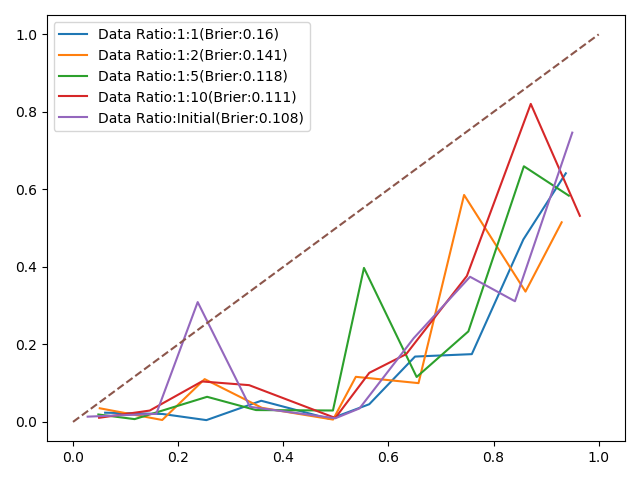


**Supplementary​ ​Figure S2**.​ ROC curve of five models.


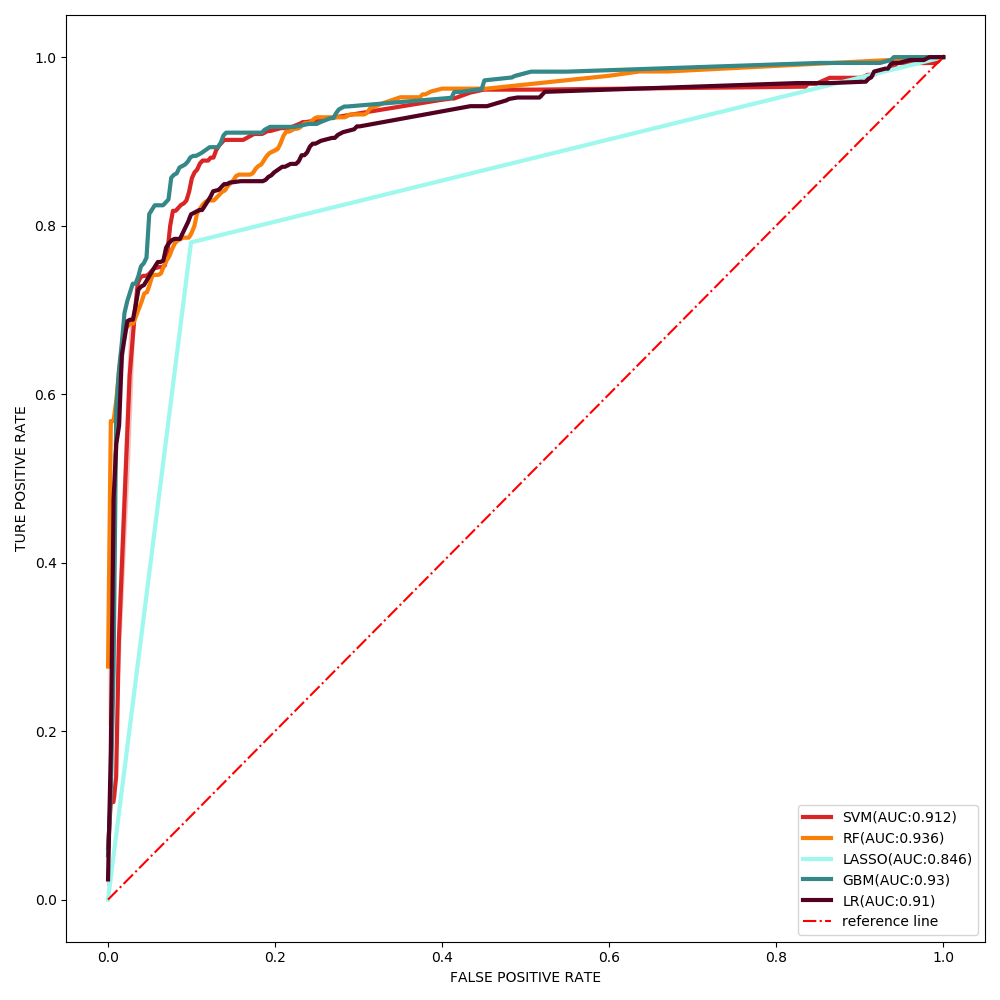


**Supplementary​ ​Figure S3**.​ PR curve of five models.


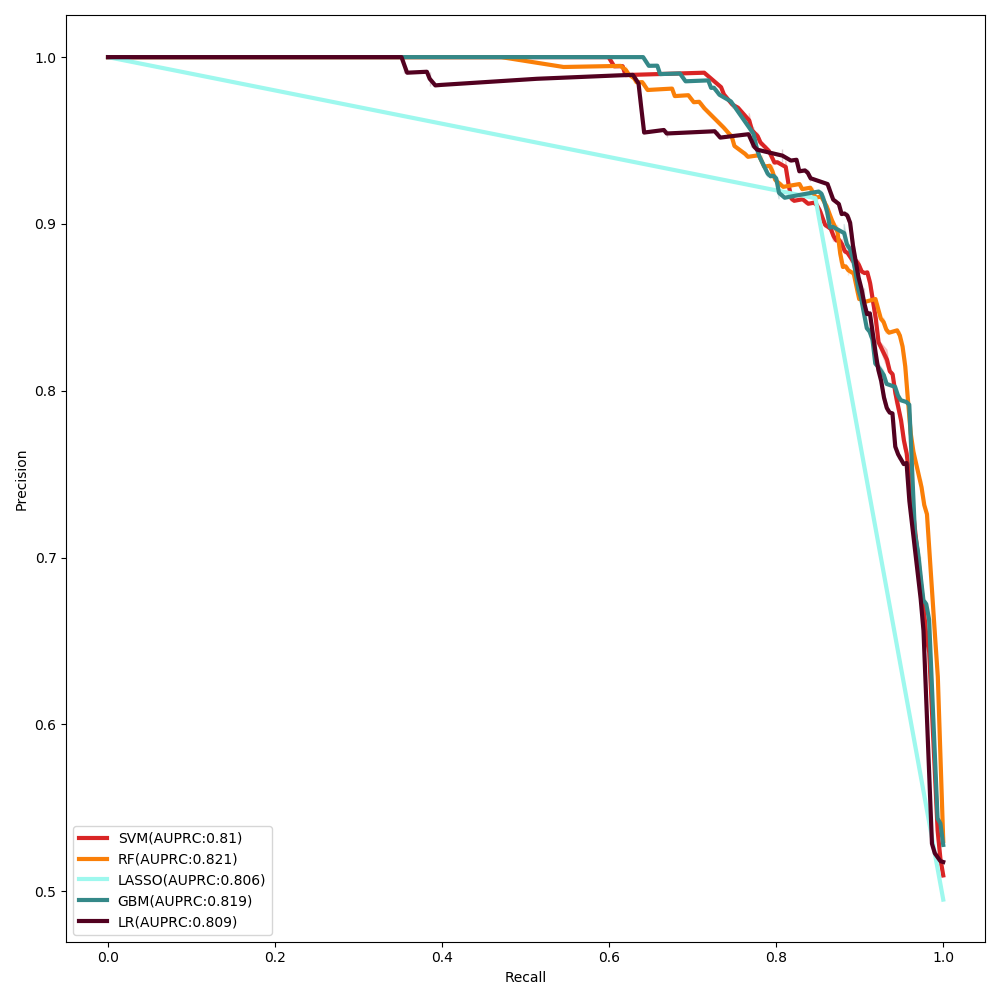

Supplement: Multimedia Appendix 1 [file medinform_v11i1e38590_app1.docx]
